# Effect of Phase Decomposition and Elemental Partitioning on Pitting Corrosion Susceptibility in Additively Manufactured High-Entropy Alloys

**DOI:** 10.3390/ma19194150

**Published:** 2026-09-29

**Authors:** Bingqian Xu, Jiapeng Sun, Jing Zhang, Jing Han, Ying Han, Guosong Wu

**Affiliations:** 1 College of Materials Science and Engineering, Hohai University, Nanjing 210024, China; 2 Jiangsu Provincial Engineering Research Center for Structure-Function Integrated Metallic Materials for Harsh Environments, School of Materials Science and Engineering, Hohai University, Changzhou 213200, China; 3 Innovation Center for Critical Materials in Hydraulic Infrastructure Safety and Water Environment Restoration, School of Materials Science and Engineering, Hohai University, Changzhou 213200, China; 4 School of Mechanical and Electrical Engineering, China University of Mining and Technology, Xuzhou 221116, China; 5 Key Laboratory of Advanced Structural Materials, Ministry of Education, Changchun University of Technology, Changchun 130012, China

**Keywords:** high-entropy alloy, microstructure, corrosion, polarization

## Abstract

Additive manufacturing generally endows high-entropy alloys (HEAs) with distinctive microstructures and excellent properties unattainable by conventional processing. This study elucidates how annealing-induced phase decomposition and elemental partitioning govern pitting corrosion behavior in a laser-directed energy-deposited (LDED) Al_0.5_CoCrFeNi HEA. Notably, the rapid LDED solidification process produces a metastable B2 phase with anomalously high Cr content in this HEA compared to its conventional manufacturing counterpart. Upon annealing at 650 °C for 1 h, (Cr, Fe, Co)-enriched *σ* and (Cr, Fe)-enriched body-centered cubic (BCC) phases preferentially precipitate within the B2 phase, concurrently depleting Cr and enriching Al in the residual B2 matrix. This elemental repartitioning critically degrades the protective capability of the B2 phase’s passive film, thereby compromising the alloy’s overall pitting corrosion resistance, as evidenced by decreased pitting and repassivation potentials. These findings advance the fundamental understanding of corrosion mechanisms in additively manufactured HEAs and offer a mechanistic rationale for designing corrosion-resistant HEAs via controlled phase transformation.

## 1. Introduction

High-entropy alloys (HEAs), defined by the presence of five or more principal elements in near-equimolar ratios, have garnered considerable research owing to their exceptional mechanical properties, corrosion resistance, and thermal stability, rendering them well-suited for application in extreme service environments [1,2,3]. Among them, multi-phase HEAs offer enhanced compositional flexibility and microstructural tunability compared to single-phase HEAs, enabling enhancement in mechanical and other key properties [4,5,6,7,8]. For example, the AlCoCrFeNi_2.1_ eutectic HEA, which is characterized by a lamellar dual-phase structure comprising ordered B2 and face-centered cubic (FCC) phases, generally demonstrates a combination of high strength and appreciable ductility [9,10]. Nevertheless, the complexity arising from multi-element compositions, diverse phase formations, and the intricate microstructure of the multi-phase HEAs also presents substantial scientific challenges.

A particular issue is the unresolved relationship among chemical composition, phase constituents, and corrosion behavior and the underlying mechanism for the multi-phase HEAs [11]. The CoCrFeNi base system, featuring a single-phase FCC structure, exhibits high ductility but limited strength. Alloying with Al drives microstructural evolution: increasing Al content induces a transformation from FCC to an FCC+ body-centered cubic (BCC) dual-phase microstructure and, at higher Al concentrations, to a single-phase BCC structure, concomitant with enhanced strength [12,13,14]. Yet, the corresponding effects on corrosion behavior remain ambiguous and often contradictory. Shi et al. observed that elevated Al levels promoted formation of a Cr-depleted BCC phase, which produced a thicker yet more defective passive film enriched with Al_2_O_3_, thereby accelerating localized corrosion of Al_x_CoCrFeNi HEAs [15,16]. Conversely, Huang et al. observed that increasing Al content in Al_x_CoCrFeNi (x = 0, 0.1, 1.0) HEAs enhanced their corrosion resistance in supercritical water [17]. Additionally, although intermetallic precipitates such as σ and L1_2_ phases are commonly present in this type of HEA and known to influence corrosion, their specific roles are still not thoroughly understood.

Unlike conventional casting, additive manufacturing (AM) techniques, notably selective laser melting (SLM) and laser-directed energy deposition (LDED) [18,19], enabling fabrication of HEAs through rapid cooling in a layer-by-layer mode [20,21,22]. This process results in non-equilibrium microstructures, especially in multi-phase HEAs [23,24], thereby introducing unique corrosion behavior. Zhou et al. reported that the AlCoCrFeNi_2.1_ HEA produced by SLM demonstrates superior corrosion resistance compared to its as-cast counterpart in saline environments [25]. Zhang et al. found that among AlxCoCrFeNi HEAs fabricated via laser melting deposition with Al content varying from 5 to 20 mol.%, the HEA with 10 mol.% Al exhibited the highest corrosion resistance [26]. This composition corresponds to a three-phase microstructure consisting of FCC, BCC, and A2 phases. In contrast, the BCC phase is generally considered to undergo preferential dissolution in NaCl solution owing to micro-galvanic corrosion between the BCC and FCC phases [27], thereby compromising the overall corrosion resistance of Al-Co-Cr-Fe-Ni HEAs.

The distinctly different corrosion behavior of AM versus conventionally fabricated HEAs, along with the high variability in the corrosion response of multi-phase HEAs, suggests that factors beyond phase constitution may critically govern the corrosion behavior of multi-phase HEAs. The constituent phases in HEAs often have a composition range rather than a fixed composition ratio [28]. Processing routes, thermal history, and phase transformations can significantly alter phase constituents and induce elemental repartitioning, thereby modifying the localized corrosion behavior of constituent phases and ultimately influencing the overall corrosion resistance of the alloy. Therefore, investigating corrosion evolution of multi-phase HEAs from the perspective of elemental partitioning is expected to provide new insights into the understanding of their corrosion behavior evolution and mechanism. In this work, the corrosion behavior of the Al_0.5_CoCrFeNi HEA fabricated via LDED and subsequent annealing was examined using electrochemical techniques to elucidate the effects of annealing-induced elemental partitioning on pitting corrosion behavior and its underlying mechanisms. The rapid cooling intrinsic to the LDED process promotes formation of non-equilibrium phase structures, which increases the driving force for elemental redistribution during annealing and amplifies the compositional differences among constituent phases before and after annealing, thereby providing an ideal condition for investigating element partitioning-driven corrosion migration in HEAs.

## 2. Materials and Methods

**Figure 1:**
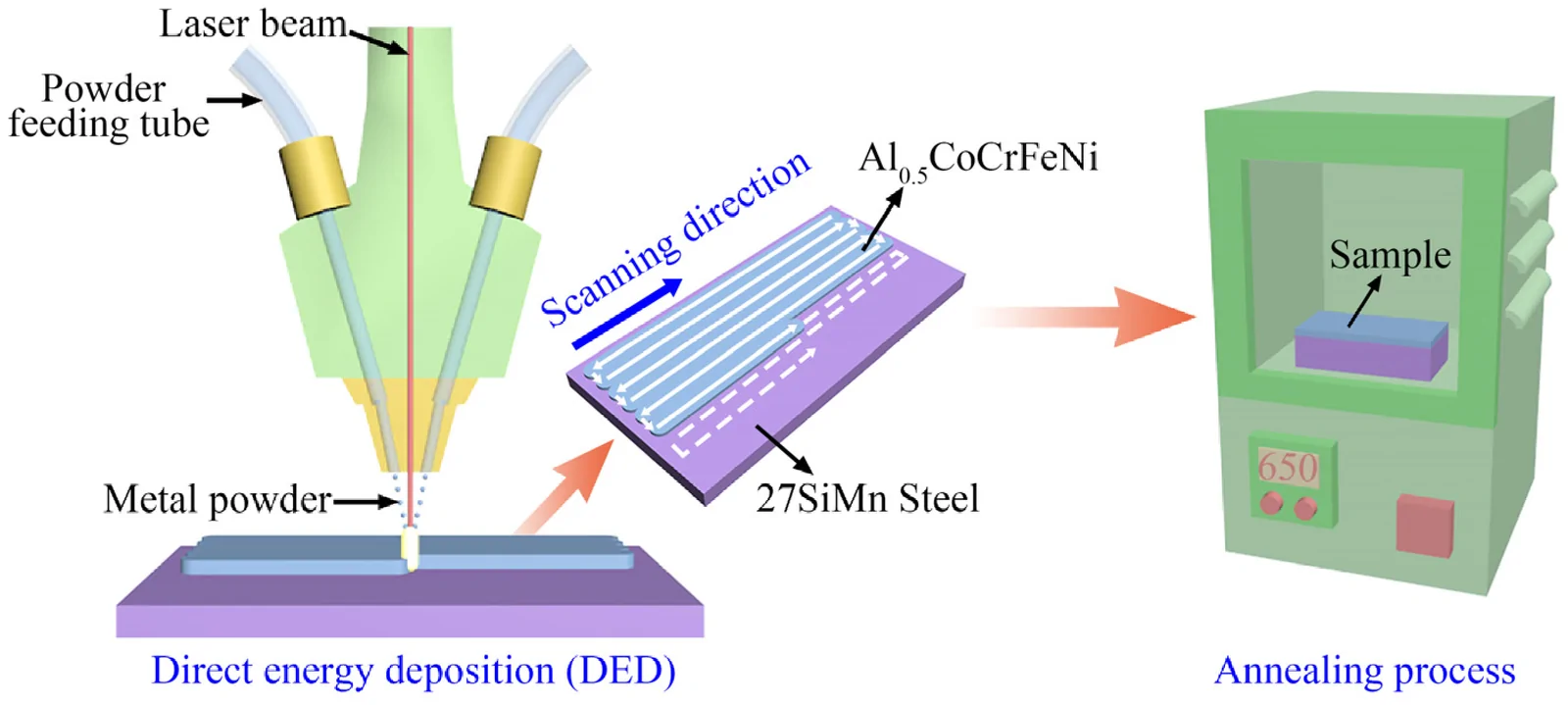
Schematic of processing method.

The HEA coating under investigation, with nominal composition Al_0.5_CoCrFeNi, was fabricated via laser-directed energy deposition (LDED) (Figure 1) and is hereafter designated the LDED sample. Gas-atomized pre-alloyed powder (Beijing Ryubon New Material Technology Co., Ltd., Beijing, China) with particle sizes ranging from 45 to 105 μm was deposited onto 27SiMn steel substrates under optimized processing parameters: laser power of 3200 W, powder feed rate of 20 g/min, scanning speed of 8 mm/s, and shielding gas flow rate of 15 L/min. An HEA coating was fabricated using this process with an approximate thickness of 1 mm. Post-deposition annealing was performed by embedding the LDED sample in graphite powder (Sinopharm Chemical Reagent Co., Ltd., Beijing, China) to prevent oxidation. The sample was heated from room temperature to 650 °C at a rate of 10 °C/min, held at 650 °C for 1 h, and then furnace-cooled to room temperature.

Microstructural characterization of the investigated samples was performed utilizing multiple complementary techniques: scanning electron microscopy (SEM, ZEISS GeminiSEM 300, Germany) coupled with energy-dispersive X-ray spectroscopy (EDS, Oxford Instruments Xplore 30, UK), electron backscatter diffraction (EBSD, Oxford Instruments NordlysMax3, UK), scanning transmission electron microscopy (STEM, FEI Talos F200X, USA) and its high-angle annular dark field (HAADF) mode. Samples for SEM were mechanically ground with progressively finer SiC abrasive papers (Wuyi Hengyu Instrument Co., Ltd., Jinhua, Zhejiang, China) to a final 3000-grit finish, sequentially polished with a 1.5 μm diamond suspension (Shanghai Xinbiao Testing Instrument Manufacturing Co., Ltd., Shanghai, China), and subsequently subjected to electrolytic polishing in a 10% oxalic acid (Sinopharm Chemical Reagent Co., Ltd., Beijing, China) solution. The EBSD samples underwent mechanical grinding and polishing, culminating in final surface preparation via argon ion beam polishing. For TEM observation, the samples were mechanically polished and followed by ion milling.

Corrosion behavior was evaluated by potentiodynamic polarization and cyclic potentiodynamic polarization measurements. All experiments were performed at room temperature using a Gamry Reference 600+ electrochemical workstation (Gamry Instruments, Inc., Warminster, USA). Corrosion samples were ground with progressively finer SiC abrasive papers to a 2000-grit finish, and ultrasonically cleaned in ethanol (Sinopharm Chemical Reagent Co., Ltd., Beijing, China) for 1 min. Prior to testing, each sample was immersed in a 3.5 wt.% NaCl (Sinopharm Chemical Reagent Co., Ltd., Beijing, China) aqueous solution for 2 h to establish a stable open-circuit potential (OCP). Potentiodynamic polarization measurements were conducted at a scan rate of 0.5 mV/s, sweeping from −0.35 V versus OCP to 0.8 V versus OCP. The corrosion potential (*E_corr_*), pitting potential (*E_pit_*), and corrosion current density (*I_corr_*) were determined from the potentiodynamic polarization curves using Gamry Echem Analyst version 7.8.4. *I_corr_* values were obtained by Tafel extrapolation for each polarization curve, with the anodic and cathodic branches fitted over the potential ranges from *E_corr_* to *E_corr_* − 150 mV and from *E_corr_* to *E_corr_* + 100 mV, respectively. These ranges were selected based on the linear behavior of the semilogarithmic polarization curves while excluding the passive and diffusion-limited regions. Cyclic potentiodynamic polarization measurements, conducted at the same scanning rate, were initiated at −0.35 V versus OCP, reversed at an apex anodic current of 5 mA/cm^2^, and returned to the initial potential. To understand the corrosion mechanism, corrosion morphologies after potentiodynamic polarization measurements were examined by SEM. Complementary analysis of the passive film chemistry and valence states was performed by X-ray photoelectron spectroscopy (XPS, Escalab 250Xi+, Thermo Fisher Scientific, USA). Before tests, the samples were immersed for 168 h. The binding energies were charge-corrected using adventitious carbon C 1s at 284.8 eV as the reference.

## 3. Results

### 3.1. Microstructure

**Figure 2:**
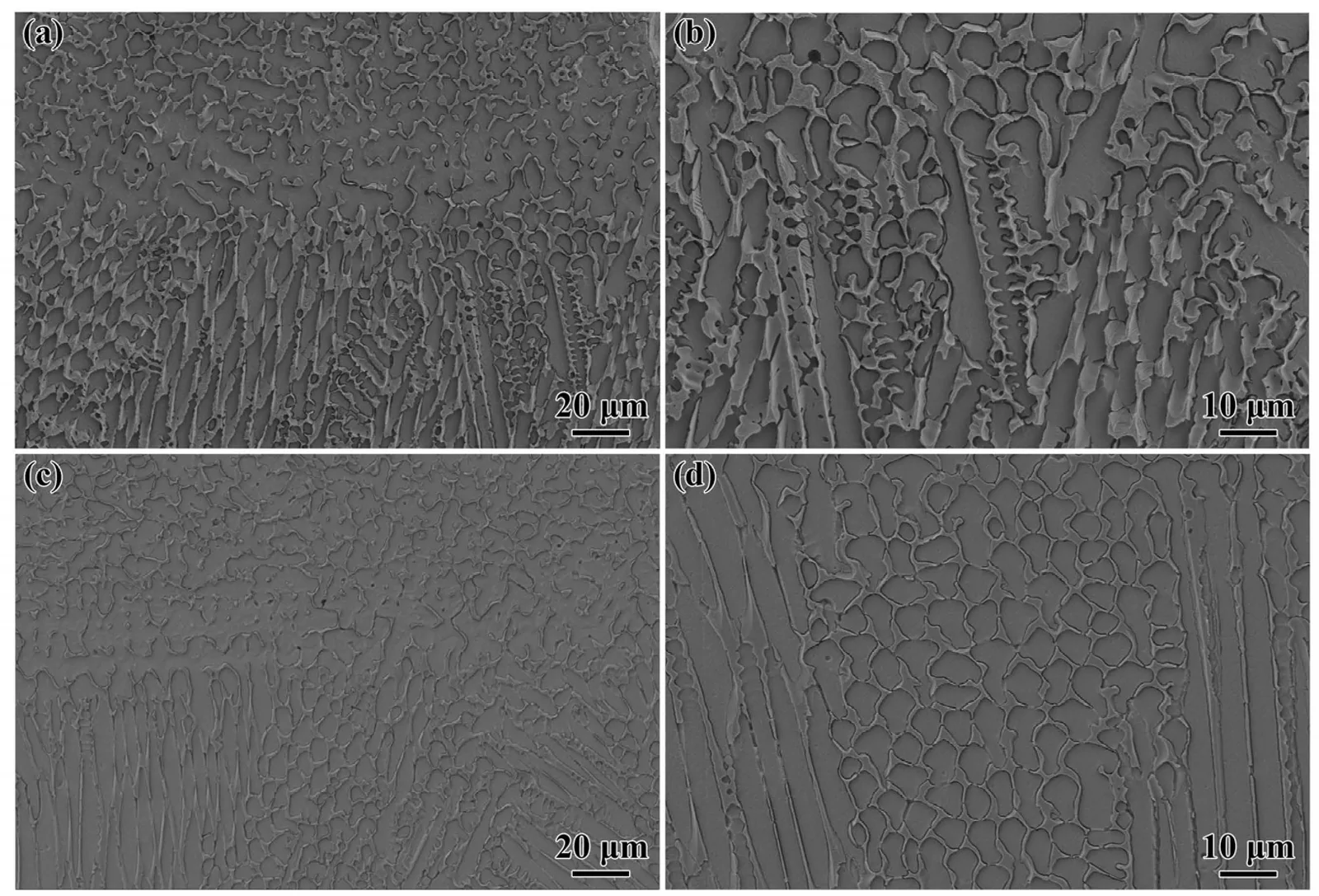
SEM micrographs of the (**a**,**b**) LDED and (**c**,**d**) annealed samples.

**Figure 3:**
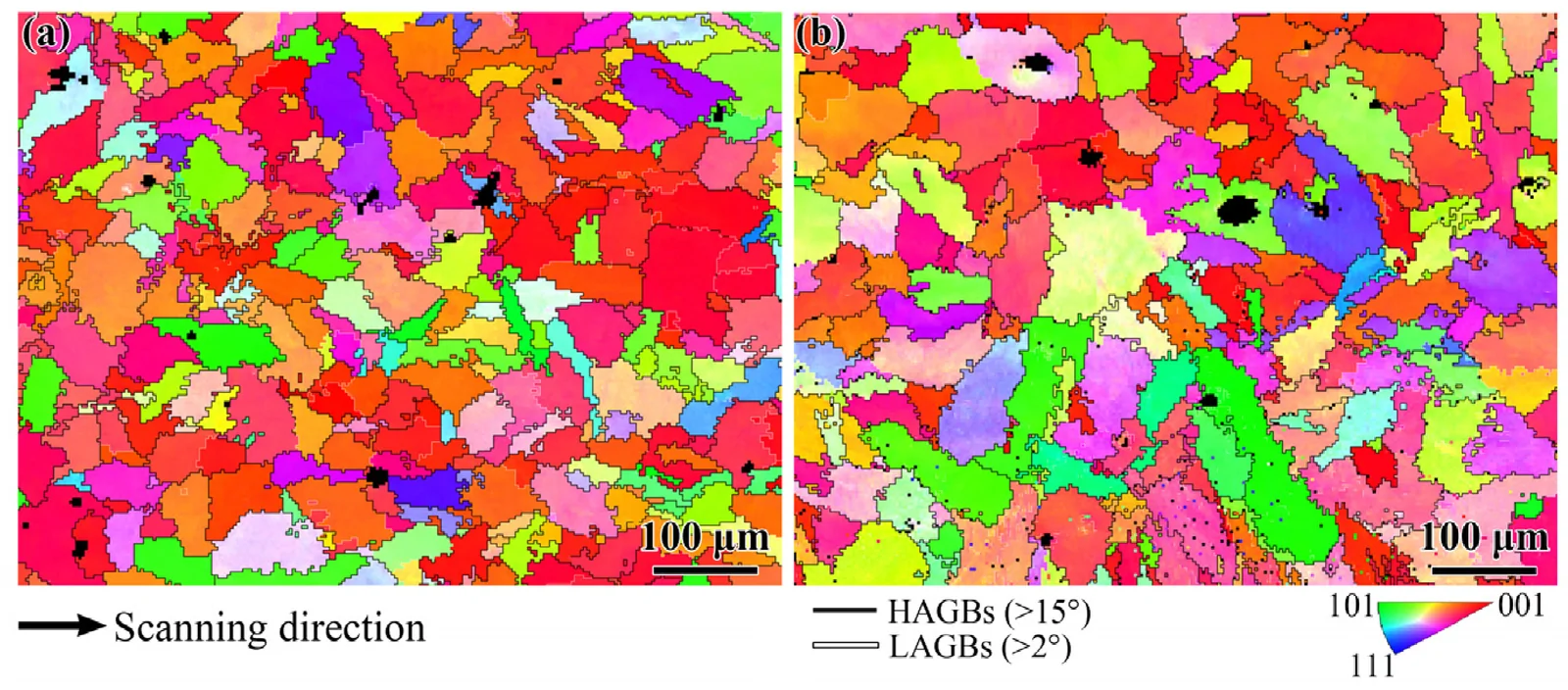
Inverse pole figure (IPF) maps of the (**a**) LDED and (**b**) annealed samples.

Figure 2 depicts SEM micrographs of the LDED and annealed samples. Both exhibit a dendritic structure, and no significant differences were observed between them. This result confirms dendritic solidification of the HEA during LDED processing. Figure 3 presents EBSD results of the LDED and annealed samples, indicating a similar coarse grain structure. The mean grain size is 41.0 μm and 43.0 μm for the LDED and annealed samples respectively, measured statistically using the intercept method.

**Figure 4:**
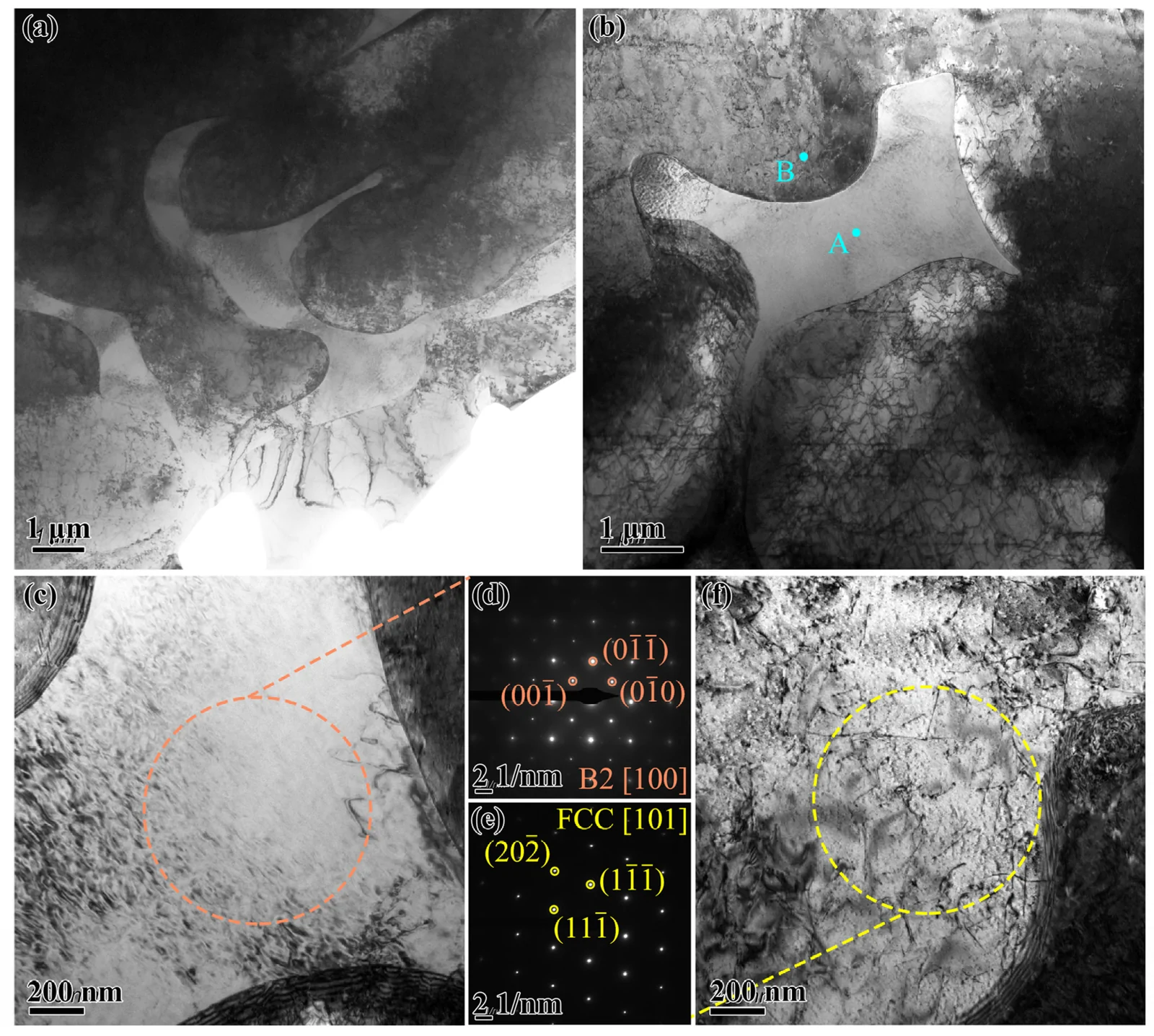
TEM micrographs of the LDED sample: (**a**,**b**) low-magnification images, (**c**,**f**) high-magnification images of the B2 phase and the FCC matrix and (**d**,**e**) corresponding SAED pattern highlighted in panel (**c**,**f**). The letters A and B in (**b**) denote the EDS analysis points, and the corresponding EDS results are listed in Table 1.

Figure 4 depicts TEM images of the LDED sample, revealing a coarse matrix within which abundant dendritic secondary phases are embedded. Selected area electron diffraction (SAED) patterns (Figure 4d,g) and EDS analyses (Figure 4e,h and Table 1) confirm that the matrix possesses a face-centered cubic (FCC) phase enriched in Cr, Fe, and Co, whereas the dendritic secondary phase is characterized as a body-centered cubic (BCC) phase enriched in Al and Ni. Prior studies have established that Al-Cr-Fe-Co-Ni HEAs comprise two distinct BCC phases: a disordered Fe- and Cr-rich BCC phase and an ordered Al- and Ni-rich B2 phase [29]. Consistent with this, this secondary phase is definitively identified as the B2 phase, the formation of which is directly ascribed to Al alloying. Notably, the current B2 phase exhibits a high Cr content of 21.53 at.%, significantly exceeding that of its conventionally processed counterparts [29].

**Figure 5:**
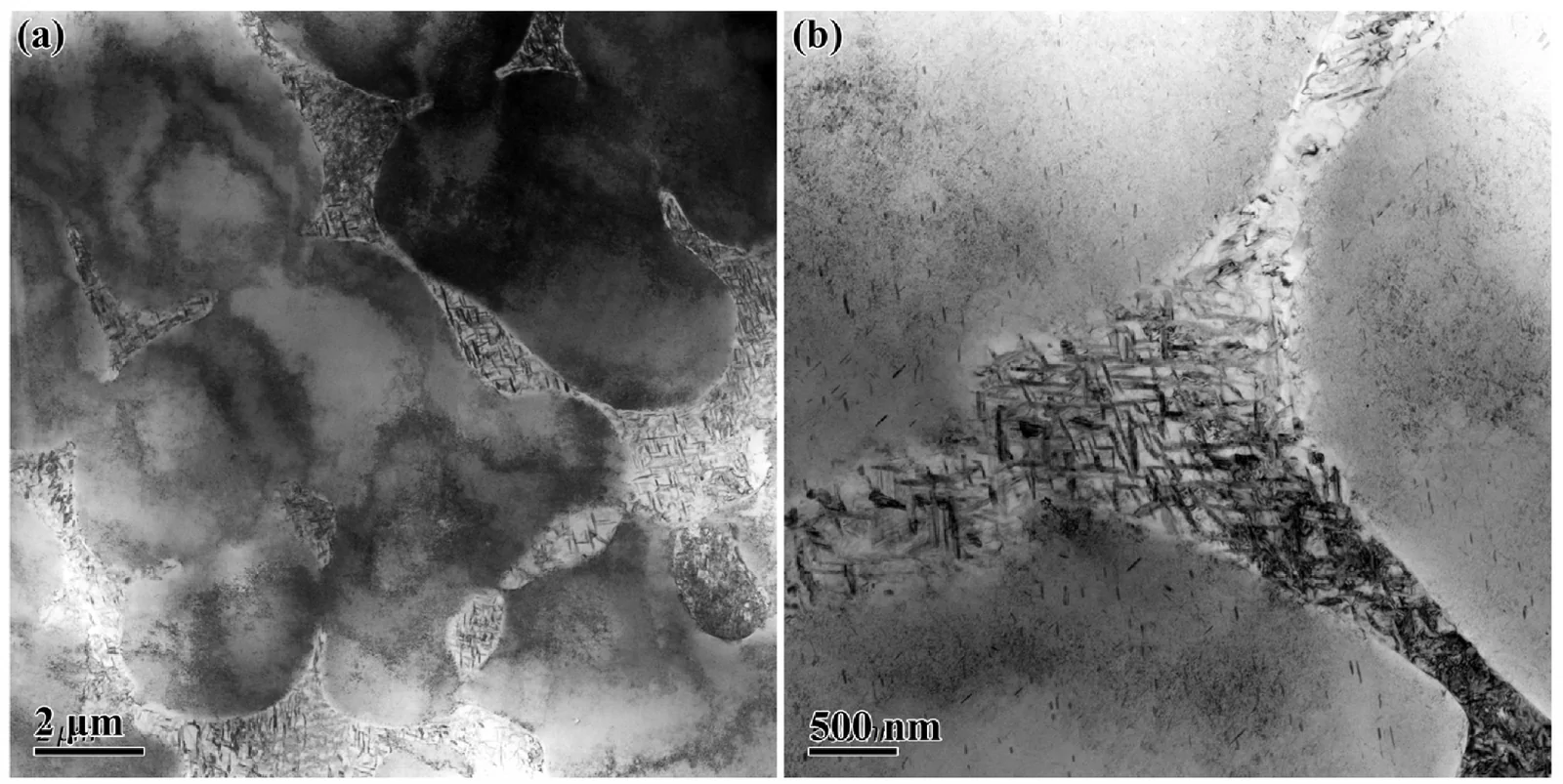
TEM images of the annealed sample: (**a**) low-magnification image, (**b**) high-magnification image.

**Figure 6:**
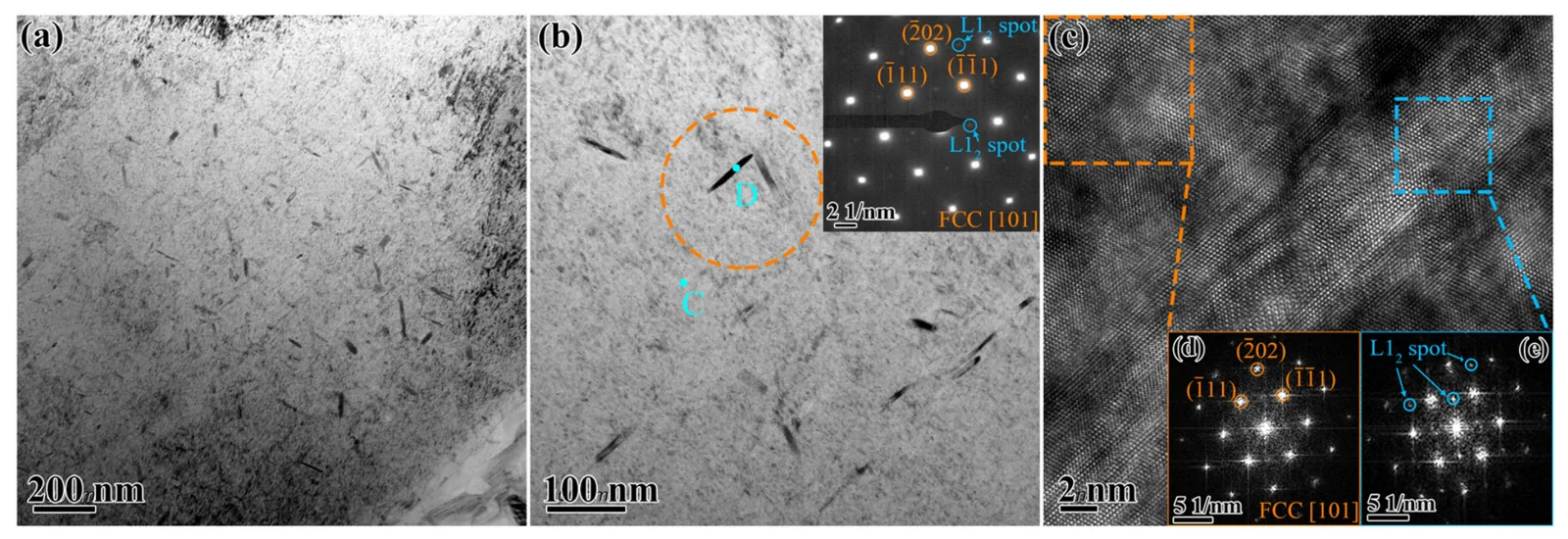
TEM micrographs of L1_2_ phase in the annealed sample: (**a**,**b**) TEM bright field images and corresponding SAED pattern, (**c**) HRTEM image, FFT pattern of (**d**) FCC matrix and (**e**) L1_2_ phase.

**Figure 7:**
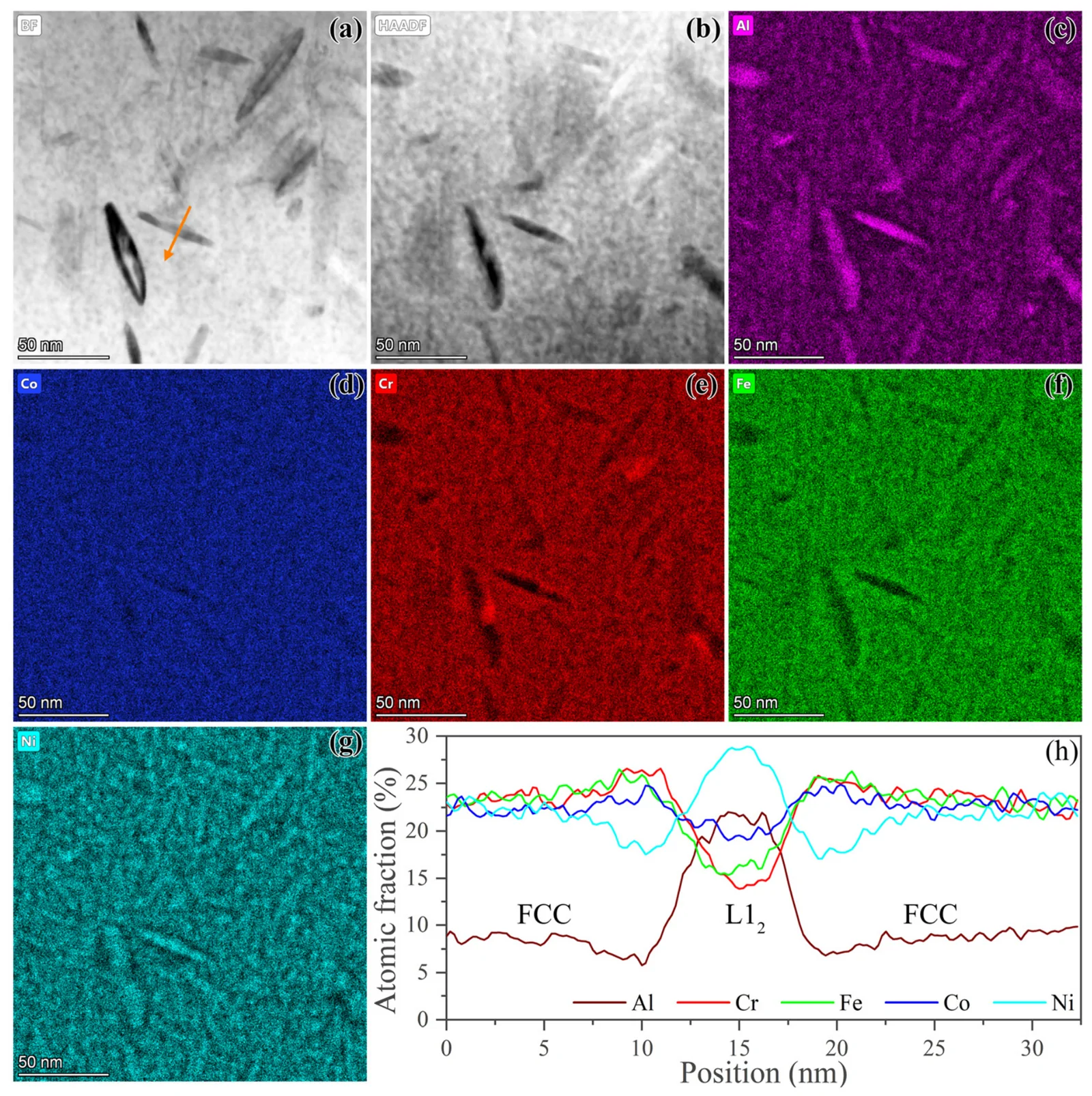
Morphology and composition of the L1_2_ phase in the annealed sample: (**a**) STEM micrograph, (**b**) paired HAADF image, (**c**–**g**) corresponding EDS mapping, (**h**) EDS line scan corresponding to the orange arrow highlighted in panel (**a**).

Figure 5 presents low-magnification TEM micrographs of the annealed sample. It is evident that a high density of nanoprecipitates is present in the B2 phase, while the FCC phase contains relatively sparse nanoprecipitates. In the FCC matrix, the nanoprecipitates are plate-like with an average thickness of 7.77 ± 2.14 nm, as shown in Figure 6a,b. Figure 6c exhibits high-resolution TEM (HRTEM) images of these nanoprecipitates. In contrast to the fast Fourier transform (FFT) pattern of the FCC matrix recorded along the [101]_fcc_ zone axis, the FFT pattern of the nanoprecipitates reveals additional {100} and {110} superlattice reflections. This distinct diffraction feature demonstrates the classification of these nanoprecipitates as the L1_2_ ordered phase. Furthermore, Figure 7 depicts the STEM and HAADF images, and corresponding EDS analysis of the L1_2_ phase. Relative to the FCC matrix, the L1_2_ nanoprecipitates are enriched in Al and Ni and depleted in Co, Cr, and Fe.

**Figure 8:**
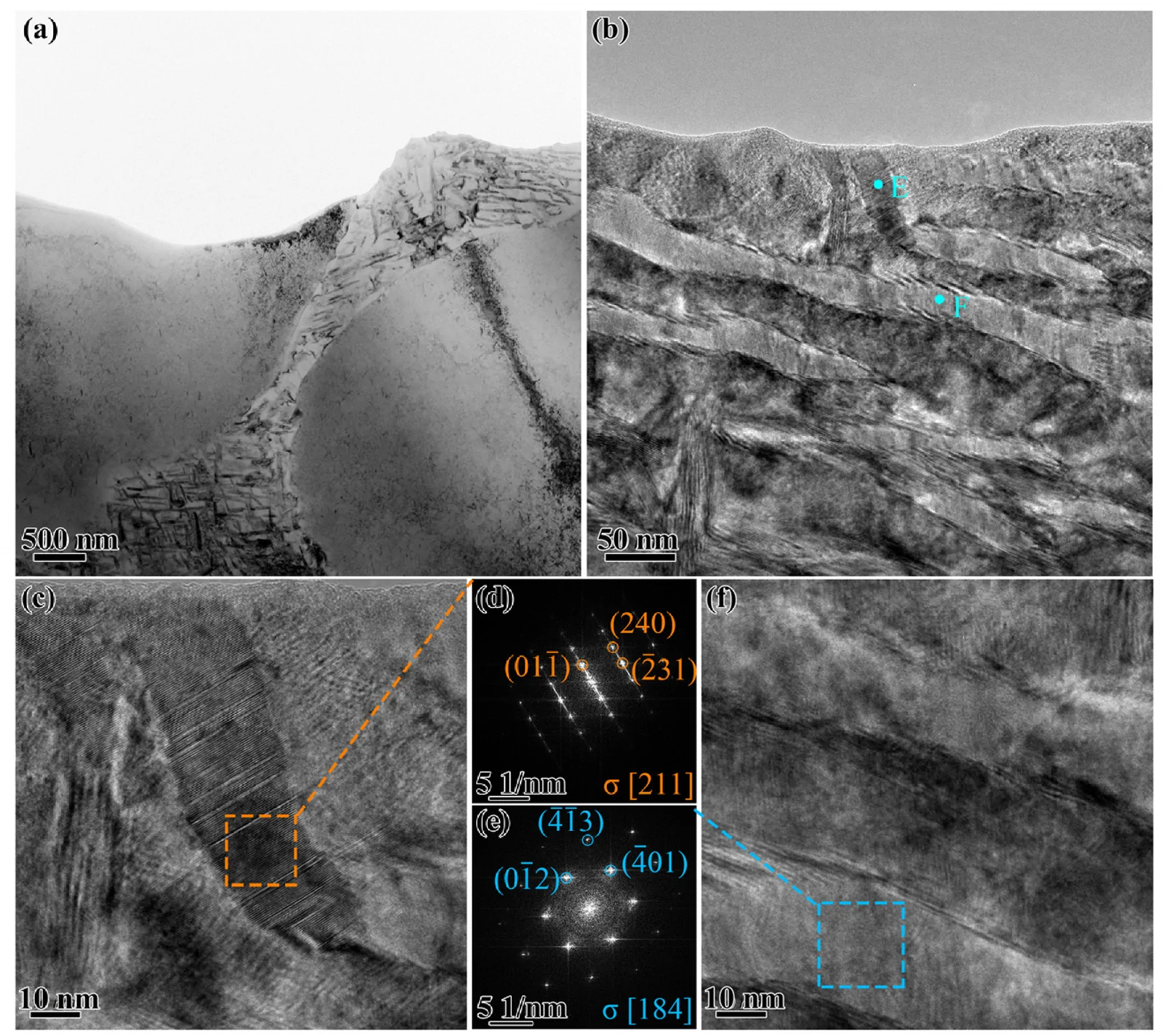
TEM micrographs of B2 and *σ* phases in the annealed sample: (**a**,**b**) TEM bright field images, (**c**,**f**) HRTEM images, (**d**,**e**) FFT patterns. highlighted in panel (**c**,**f**).

**Figure 9:**
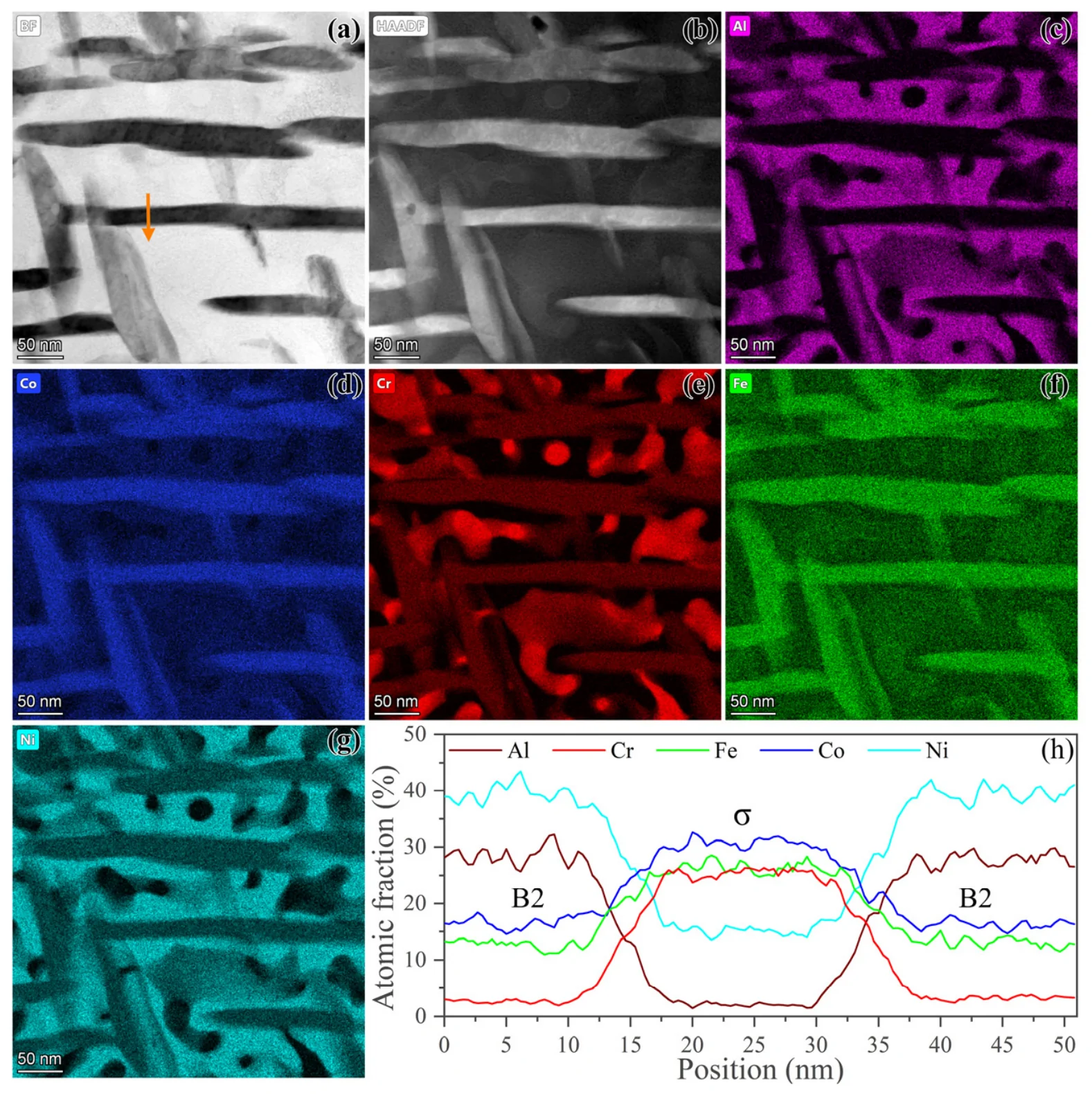
Morphology and composition of a typical region including B2, *σ* and (Cr, Fe)-enriched BCC phases in the annealed sample: (**a**) STEM image, (**b**) corresponding HAADF image, (**c**–**g**) corresponding EDS mapping, and (**h**) EDS line scan corresponding to the orange arrow highlight in panel (**a**).

Figure 8 highlights the nanoprecipitates within the B2 phases of the annealed sample. These nanoprecipitates exhibit a rod-like morphology and are primarily distributed along two distinct orientations. They have an average thickness of 22.79 ± 6.10 nm, which is much larger than the L1_2_ nanoprecipitates inside the FCC phase. The FFT patterns confirm all these nanoprecipitates along two distinct orientations as the *σ* phase, as indicated in Figure 8d,g. Figure 9 shows the STEM and HAADF micrographs and corresponding EDS analysis result of a typical B2 phase region. The EDS results prove that the *σ* phase is enriched in Fe, Co, and Cr elements and depleted in Al and Ni elements compared to the B2 phase. In addition, the HAADF image (Figure 9b) and EDS mapping (Figure 9c–g) show the presence of another nanoprecipitate enriched in Cr and Fe elements within the B2 phase. Based on its distinct compositional profile and consistent with previous studies on this alloy system [30], these precipitates are identified as the A2 phase, i.e., a disordered BCC phase. This phase is inferred to possesses a disordered BCC structure with lattice parameters comparable to the B2 phase, making them challenging to distinguish, while its distinct compositional profile enables clear differentiation from the B2 phase. Notably, Cr content in the B2 phase decreased to approximately 3 at.% due to the annealing-induced precipitation of *σ* and (Cr, Fe)-enriched BCC phases, which is significantly lower than the value of 21.53 at.% observed in the LDED sample.

### 3.2. Corrosion Behavior

**Figure 10:**
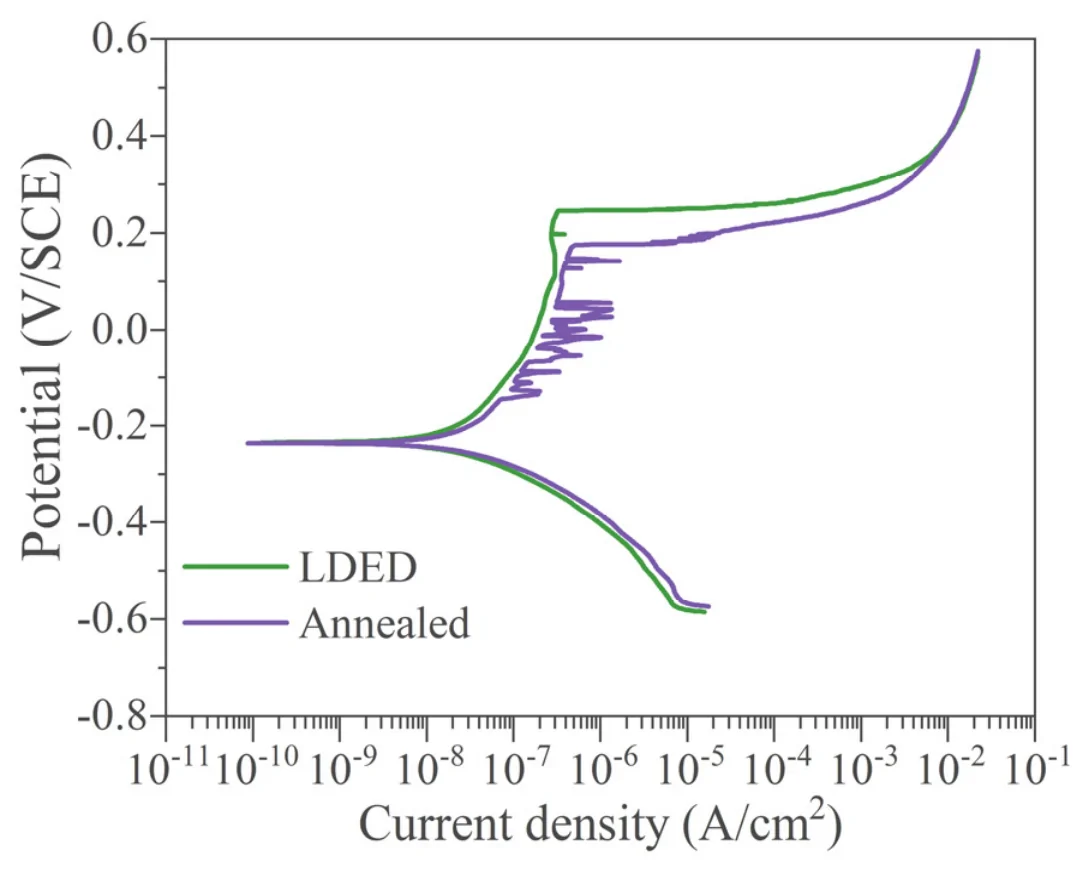
Potentiodynamic polarization curves.

Figure 10 depicts potentiodynamic polarization curves for the LDED and annealed samples, while Table 2 lists the extracted open-circuit potential (*E_ocp_*), corrosion potential (*E_corr_*), pitting potential (*E_pit_*), and corrosion current density (*I_corr_*) determined by Tafel extrapolation. Comparable anodic and cathodic polarization curves are observed for LDED and annealed samples. Their anodic polarization curves exhibit well-defined passive regions without active-to-passive transition, signifying inherent self-passivation behavior for both samples. Despite exhibiting similar *E_corr_*, the annealed sample displays an elevated corrosion current density (*I_corr_* = 174 nA) compared to its LDED counterpart (*I_corr_* = 140 nA). The significantly higher *I_corr_* demonstrates that the annealing process degrades the corrosion resistance inherent to the LDED sample. Comparative analysis of pitting potentials reveals that the annealing treatment reduces *E_pit_* by 35% (from 254 mV for to 165 mV). *E_pit_* represents the critical potential at which the passive film undergoes rupture, initiating stable pitting corrosion. Higher *E_pit_* values correspond to greater resistance to pitting corrosion. Consequently, a reduction in *E_pit_* indicates that the annealing process diminishes the pitting corrosion resistance of the LDED Al_0.5_CoCrFeNi HEA. Compared with recently developed AlCoCrFeNi alloys, the alloys investigated in this work exhibit enhanced corrosion resistance, particularly the LDED Al_0.5_CoCrFeNi HEA. Moreover, metastable pitting events, characterized by transient anodic current peaks within the passive region, are observed in both samples. Notably, these events exhibit higher frequency and earlier initiation in the annealed sample compared to its LDED counterpart. It should be noted that the scan rate of 0.5 mV/s may not capture all metastable pitting events; however, since both samples were measured under identical conditions, the relative comparison of metastable pitting activity between the LDED and annealed samples remains valid.

**Figure 11:**
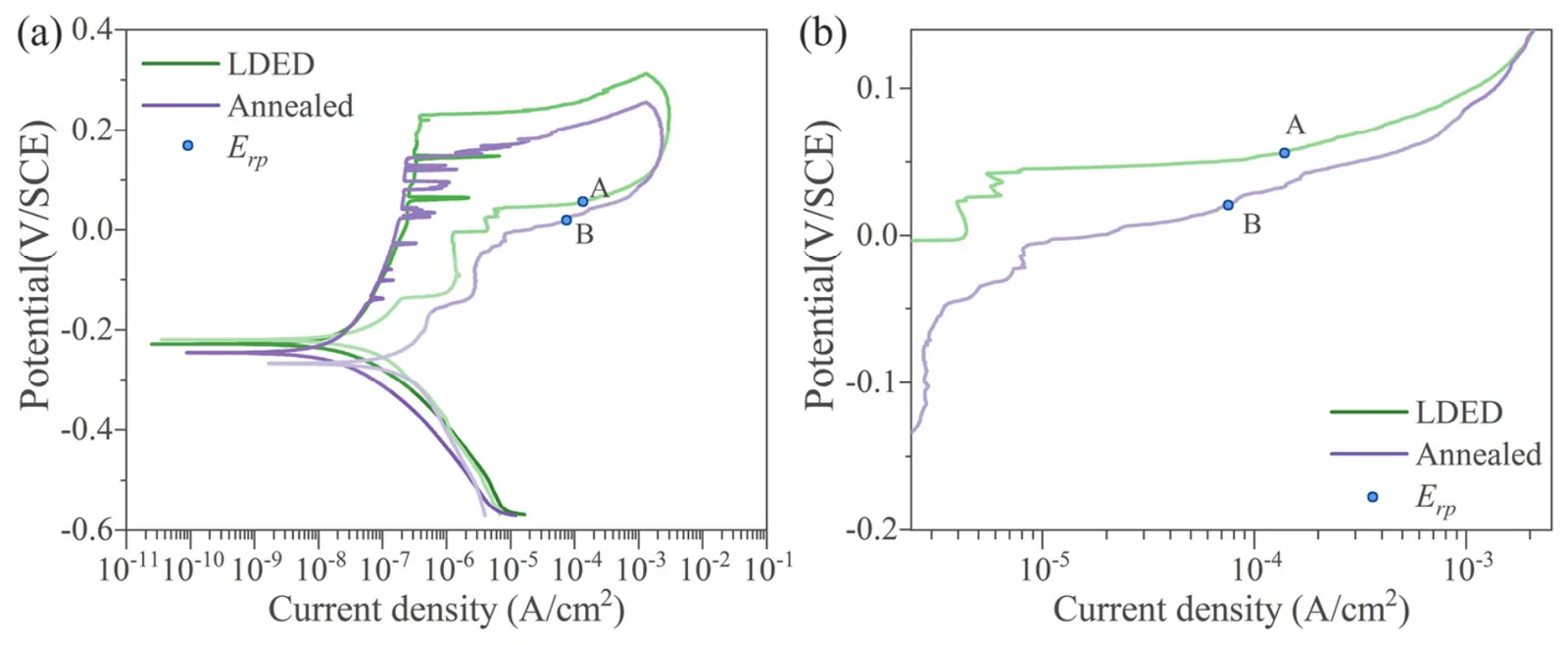
(**a**) Cyclic potentiodynamic polarization curves and (**b**) the corresponding locally magnified views.

Cyclic potentiodynamic polarization measurements were carried out to evaluate the self-repair capacity of the passive film, with results presented in Figure 11. Both LDED and annealed samples exhibit positive hysteresis loops, evidenced by higher reverse scan current densities relative to forward scanning. This behavior confirms susceptibility to pitting corrosion in both samples. Conventionally, loop closure between reverse and forward polarization scans within the passive region signifies the occurrence of repassivation at the pit bottom, with the corresponding potential defined as the repassivation potential to evaluate repassivation capability. Here, among triplicate measurements for the LDED sample, the reverse and forward scan polarization curves intersect within the passive region in two cases. In contrast, only one measurement for the annealed sample demonstrates passive region loop closure. Consequently, quantitative assessment of repassivation capability evolution between two investigated samples remains challenging.

Notably, all the reverse scan curves across both samples exhibit a finite passive region with larger passive current density compared to the forward scan curves. This finding demonstrates sustained repassivation capacity in both the LDED and annealed samples. This phenomenon likely originates from highly acidic solution entrapment within pits, where geometric confinement restricts solute diffusion, thereby inhibiting chemical restoration to pre-pitting conditions during reverse scanning. Consequently, repassivation potential *E_rp_*, defined as the potential at which the current density exhibits a precipitous change, as indicated by the blue dots in Figure 11, is used to quantitatively assess the repassivation capability. This definition, originally established for stainless steels, has been rigorously validated [37]. The LDED sample exhibits an *E_rp_* value of 56.5 mV, compared with 2.1 mV for the annealed sample. These results establish that the annealing degrades the repassivation capability of the LDED Al_0.5_CoCrFeNi HEA.

### 3.3. Passive Film Characterization

**Figure 12:**
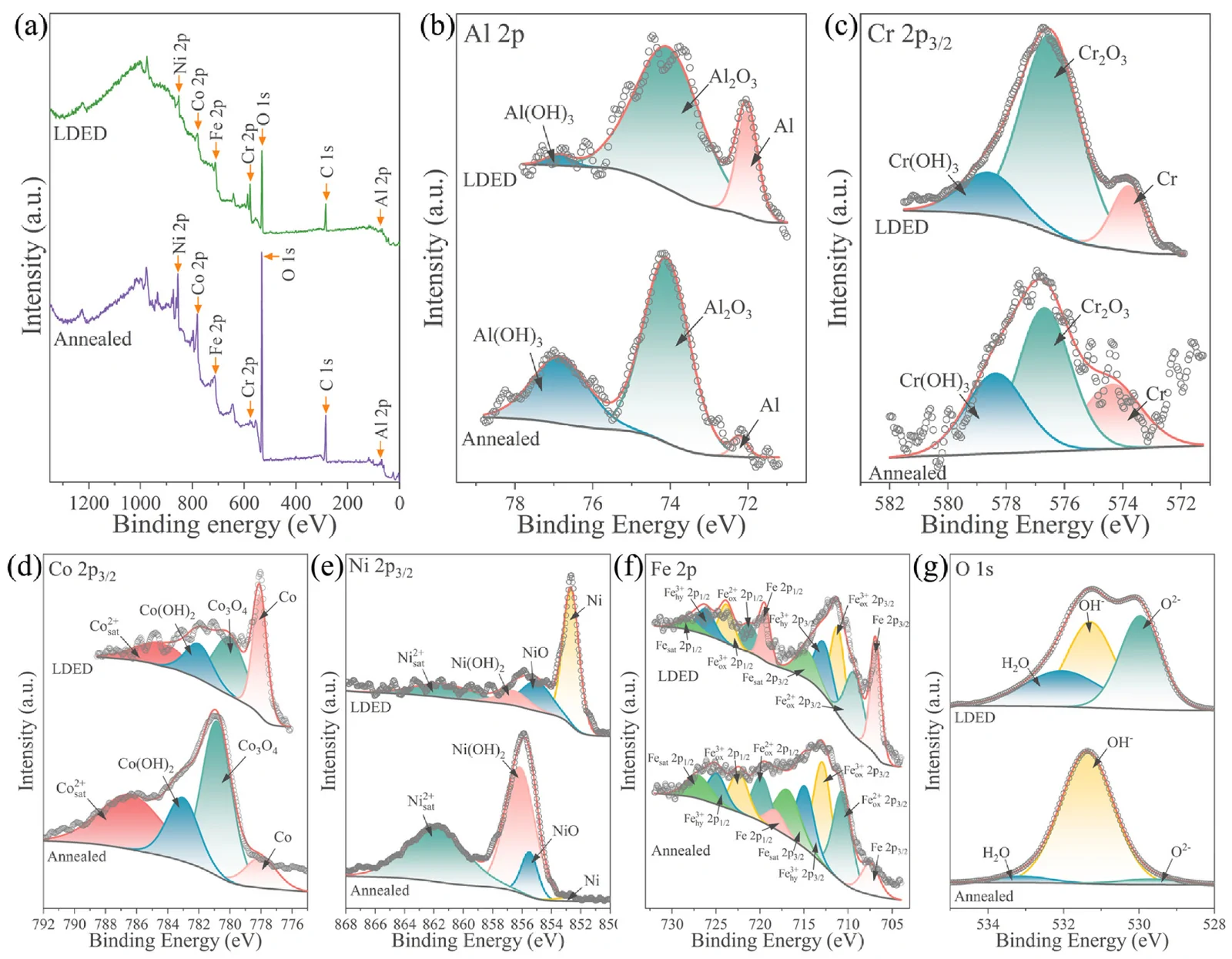
XPS results of the LDED and annealed samples after 168 h immersion: (**a**) survey spectra, and high-resolution spectra of (**b**) Al 2p, (**c**) Cr 2p_3/2_, (**d**) Co 2p_3/2_, (**e**) Ni 2p_3/2_, (**f**) Fe 2p, (**g**) O 1s.

The chemical compositions and valence states of the passive films that developed on both the LDED and annealed samples after 168 h of immersion were characterized by XPS. An immersion duration of 168 h was selected to ensure the passive film attained a steady-state condition. As exhibited in the survey spectra (Figure 12a), the O 1s peak appears as the most intense peak relative to the background in both samples. The O 1s spectrum (Figure 12g) is split into three peaks corresponding to O^2−^, OH^−^, and a small amount of H_2_O, indicating that the passive films in both alloys consist primarily of oxides and hydroxides. The Al 2p, Co 2p_3/2_, Ni 2p_3/2_, and Fe 2p spectra are all decomposed into their corresponding metallic, oxide, and hydroxide species for both samples, as shown in Figure 12b–f. For example, the Cr 2p_3/2_ spectrum (Figure 12c) is separated into metallic Cr, Cr_2_O_3_, and Cr(OH)_3_.

**Figure 13:**
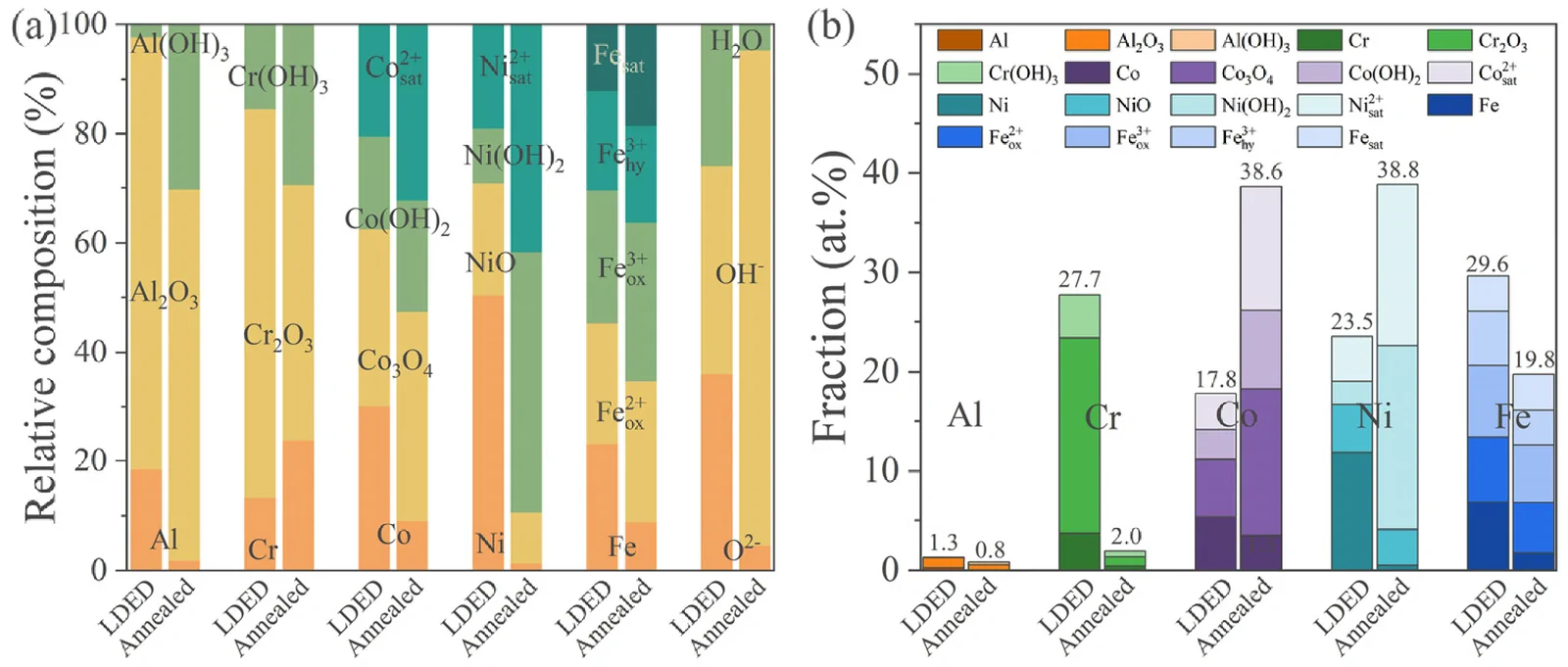
(**a**) The relative compositions of metallic, oxidic, and hydroxidic states of the elements and (**b**) compound fraction of the various metallic elements in the passive film, determined by XPS.

Figure 13 displays the relative compositions of metallic, oxidic, and hydroxidic species, as well as the compound distribution of various metallic elements within the passive film, as derived from XPS. In the LDED sample, Cr_2_O_3_ constitutes the predominant compound of the passive film. In contrast, the content of Cr_2_O_3_ decreases significantly in the passive film of the annealed sample, whereas the contents of Co/Ni oxides and hydroxides increase markedly. The passive films formed on both samples contain only minor amounts of Al, as indicated by the weak Al 2p signals in the survey spectra. High-resolution Al 2p spectra reveal that the residual Al in the LDED sample is present predominantly as Al_2_O_3_, with a small fraction remaining in the metallic state. In the annealed sample, the Al content dramatically declines, and the residual Al occurs as both Al_2_O_3_ and Al(OH)_3_. Moreover, the LDED sample exhibits a higher Cr_2_O_3_/Cr(OH)_3_ ratio than the annealed sample. Compact Cr_2_O_3_ is widely recognized as a key contributor to protective passive film on the surface of HEAs and stainless steels [38,39,40]. Consequently, the reduced Cr_2_O_3_ content is responsible for the diminished pitting resistance of the annealed sample.

### 3.4. Pitting Morphology

**Figure 14:**
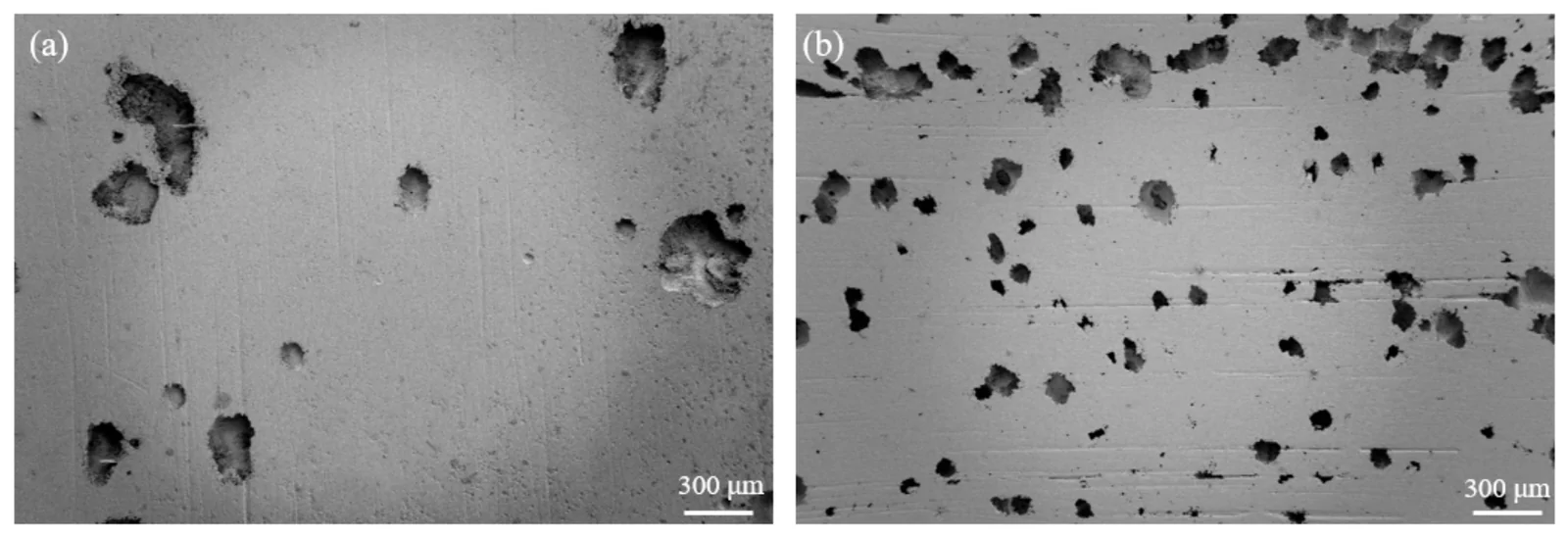
SEM analysis of corrosion morphologies after potentiodynamic polarization measurements: (**a**) LDED sample and (**b**) annealed sample.

Figure 14 exhibits the surface morphologies of the LDED and annealed samples following potentiodynamic polarization measurements, confirming localized pitting corrosion in both samples. The corrosion pit diameters in the LDED sample ranged from 90 to 400 μm, and those in the annealed samples ranged from 80 to 200 μm. The majority of corrosion pits exhibit characteristic lacy cover morphology, with no significant morphological differences between the two samples. Notably, the annealed sample displays a higher pit density compared with the LDED sample, indicative of reduced resistance to pitting corrosion.

**Figure 15:**
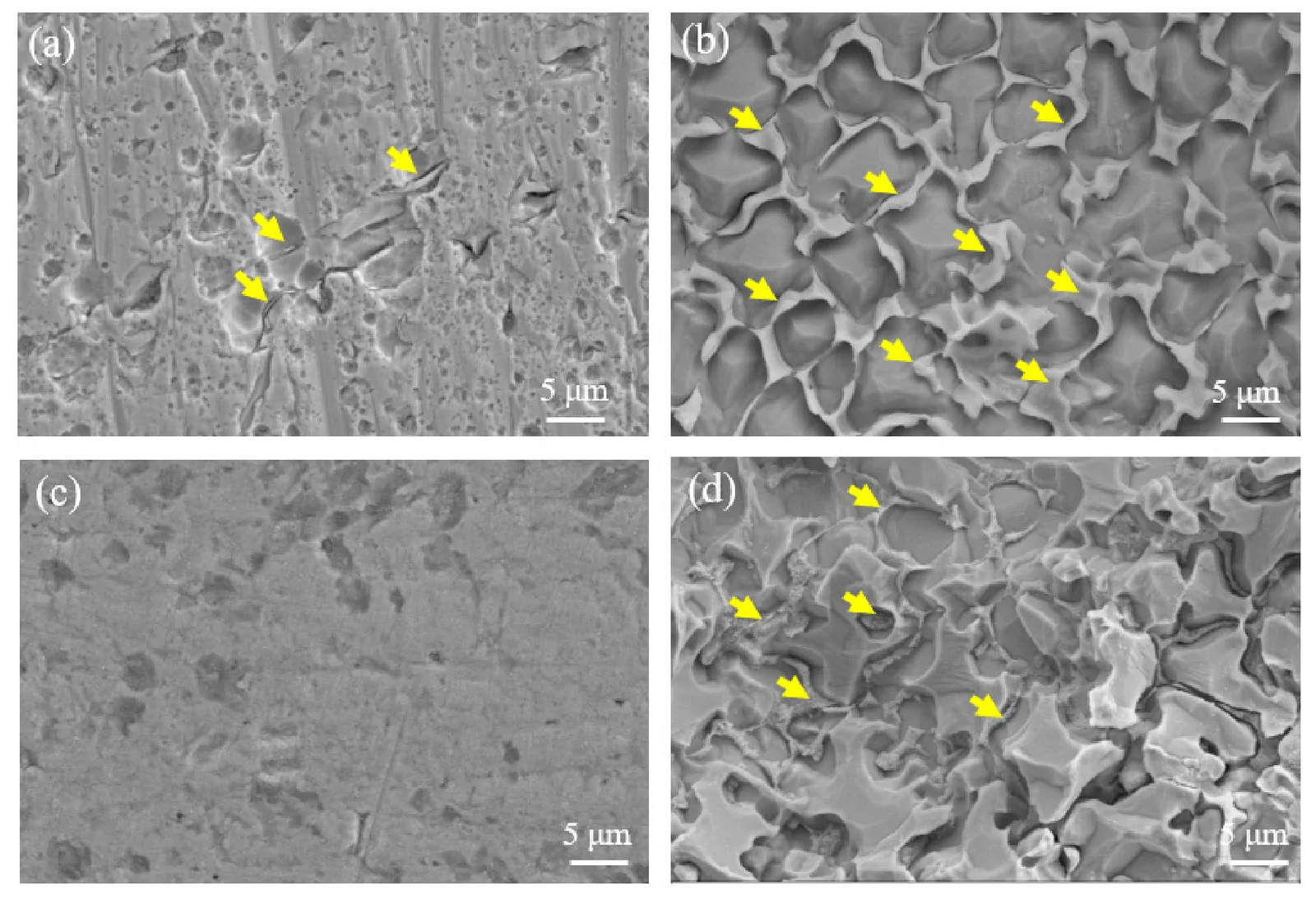
SEM analysis of corrosion morphologies after post-potentiodynamic polarization: (**a**,**b**) LDED sample and (**c**,**d**) annealed sample at (**a**,**c**) surface and (**b**,**d**) pit interiors. Yellow arrows highlight some exposed B2 phases.

Figure 15 depicts high-magnification SEM micrographs of the LDED and annealed sample surface following potentiodynamic polarization measurements, elucidating the role of nanoprecipitates in the observed corrosion behavior. Profuse micron-scale pit nucleation sites across both sample surfaces are observed, coexisting with extensive macroscopic pit development. These small pits are suggested to be metastable pits, representing the initial stages of pitting corrosion. A striking contrast exists in the morphology of metastable pits between the two samples. In the LDED sample, the metastable pits frequently form adjacent to the B2 phases, with significant corrosion observed at the B2/FCC interface. This result indicates that pitting preferentially initiates at the B2/FCC interfaces. Conversely, the annealed sample exhibits metastable pits formed through the dissolution of B2 phase domains.

Figure 15b,d depict the morphology observed at the bottom of a typical pit. In the pit interior, the passive film has ruptured, thereby triggering material dissolution via an active corrosion mechanism. For the LDED sample, dissolution of the FCC matrix exposes a protruding B2 phase. More pronounced corrosion in the B2/FCC interfacial vicinity is observed, suggesting the occurrence of micro-galvanic corrosion. For the annealed sample, B2 phase domains display distinctive selective dissolution, manifesting as porous morphology. The presence of many cavities, coupled with their morphological characteristics, indicates that they originate from the complete dissolution of B2 phase domains. This demonstrates that within pit bottoms, B2 phase domains dissolve preferentially over the FCC matrix in the annealed sample.

## 4. Discussion

### 4.1. Microstructure Formation Mechanism

**Figure 16:**
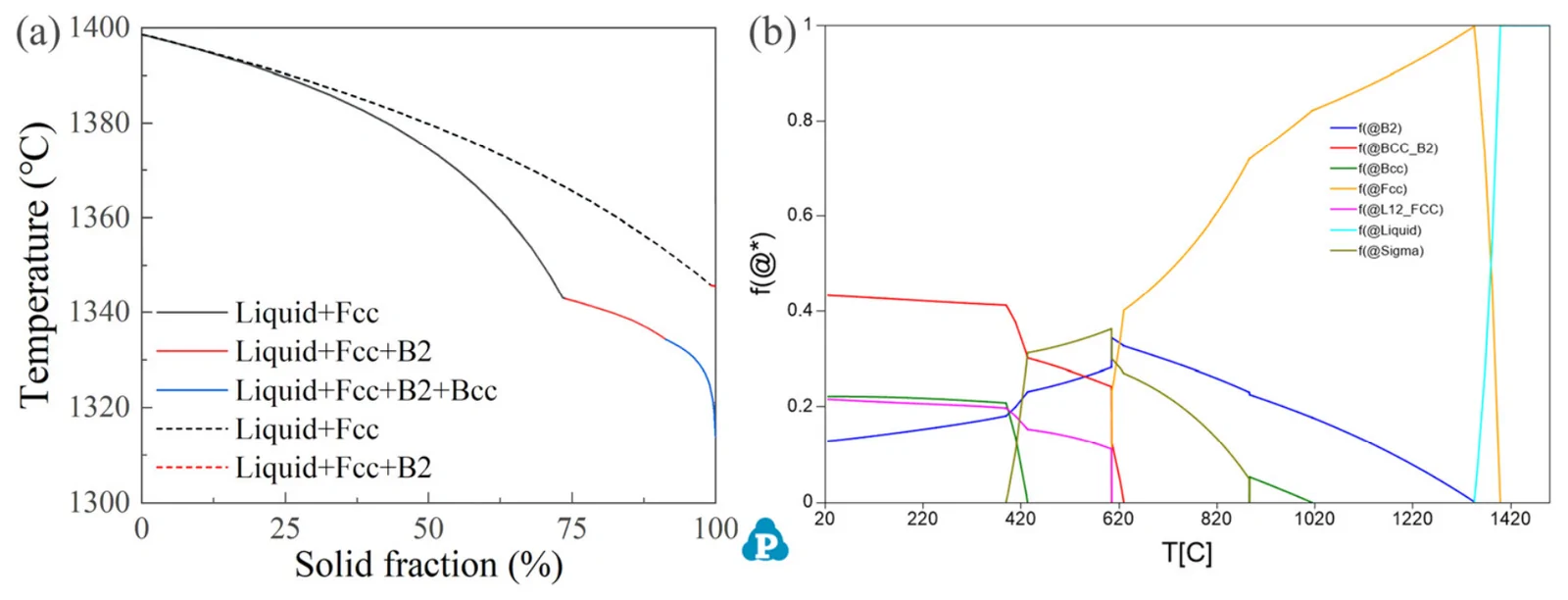
(**a**) Solidification sequence and (**b**) equilibrium phase fraction evolution with temperature for the Al_0.5_CoCrFeNi HEA, calculated using PANDAT.

Figure 16 presents PANDAT-calculated (version 2022) solidification behavior and equilibrium phase fractions for the Al_0.5_CoCrFeNi HEA. Under equilibrium solidification conditions, the FCC phase nucleates and propagates from the liquid phase, initiating transformation at 1398.65 °C. The B2 phase begins to form within the residual liquid phase at 1345.85 °C. Finally, solidification completion occurs at 1345.67 °C. The resultant microstructure exhibits a dominant FCC phase (99.84%), with a minor B2 constituent (0.16%), and a relatively narrow solidification temperature range of 52.98 °C. Notable deviations in phase fractions emerge under Scheil solidification conditions: the B2 phase fraction significantly increases to 6.08%, and an additional 1.11% BCC phase is developed during the final stages of solidification. The overall solidification temperature range expands to 84.9 °C. The ultra-high cooling rate (~10^3^–10^5^ K/s) in LDED processing establishes non-equilibrium solidification behavior [41]. Consequently, Scheil–Gulliver modeling provides closer approximation to experimentally observed phase formation than equilibrium calculations. Compared to equilibrium solidification conditions, the Scheil solidification condition leads to an expanded solidification range, thereby enhancing constitutional undercooling and more readily favoring the formation of a columnar microstructure. This also corroborates our experimental findings of a columnar microstructure in the LDED sample. The initial formation of the B2 phase occurs via a eutectic reaction from the liquid state [42]. The morphology of the B2 phase observed in the LDED sample further supports this result.

Following solidification, subsequent cooling under equilibrium conditions promotes complex solid-state reactions that precipitate *σ* (sigma) and L1_2_ phases while significantly altering phase fractions of the pre-existing B2, FCC, and BCC phases, as illustrated in Figure 16b. However, the rapid cooling rates inherent to LDED effectively suppress the majority of these solid-state transformations. Consequently, the LDED sample retains its as-solidified microstructure dominated by FCC and B2 phases. Thermodynamic calculation predicts minor BCC phase formation, but experimental characterization fails to detect its presence. This discrepancy is potentially attributed to its low content and similar lattice structure to the B2 phase.

Isothermal annealing at 650 °C for 1 h promotes solid-state phase transformations. Thermodynamic calculations indicate that the equilibrium phase assemblage at this temperature consists of FCC, BCC, and *σ* phases. However, minor BCC and L1_2_ phases were also detected during microstructural characterization of the annealed samples. The deviation between experimental observation and thermodynamic prediction is potentially explained by two mechanisms: firstly, the nucleation of metastable BCC and L1_2_ phases occurring during cooling after isothermal treatment, and secondly, inherent inaccuracies present within the utilized thermodynamic framework or its predictive capabilities.

### 4.2. Corrosion Mechanism

Electrochemical measurements confirm that the LDED Al_0.5_CoCrFeNi HEA possesses self-passivation capability, sustaining a stable passive region. Post-annealing, however, degrades both pitting resistance and repassivation capability, manifested by reduced pitting potential and repassivation potential. Microstructure characterization demonstrates that annealing promotes: (i) L1_2_ nanoprecipitate formation within the FCC matrix, and (ii) *σ* and BCC nanoprecipitates within B2 phase domains. Consequently, the observed deterioration of corrosion resistance is attributed to the formation of these nanoprecipitates. While nanoprecipitates are known to enhance alloy strength and hardness through the Orowan strengthening mechanism, the present work focuses on their adverse effect on corrosion resistance.

Our results demonstrate that the B2/FCC interface serves as the primary nucleation site for corrosion in the LDED sample, aligning with findings previously reported for Al-Co-Cr-Fe-Ni HEAs, attributable to the interface’s preferential adsorption of Cl^−^ ions accelerating passive film breakdown. Notably, while galvanic corrosion is observed between the B2 phase and FCC matrix, detailed examination reveals that the B2 phase retains its structural integrity even within substable and stable pits, indicating that galvanic corrosion is insufficient to cause its complete dissolution under current conditions, as shown in Figure 15. Contrary to prior understanding, the B2 phase—consistent with its lower corrosion potential relative to the FCC phase—behaves anodically in the B2/FCC micro-galvanic couple, resulting in its selective dissolution in cast Al-Co-Cr-Fe-Ni HEAs [15,16,26]. This result demonstrates that the present LDED process alters the corrosion behavior of the B2 phase, endowing it with markedly enhanced corrosion resistance compared to its cast counterpart.

Previous studies have proven that the B2 phase, being Al- and Ni-rich but Cr-depleted relative to the FCC matrix, inherently possesses a lower work function due to Cr’s high work function [15,16]. The low work function generally means the B2 phase is less noble than the FCC matrix, and thus is more likely to be selectively dissolved. The present study reveals that the Cr content within the B2 phase of the LDED sample reaches approximately 90% of that in the FCC matrix—significantly exceeding the Cr concentration observed in the cast HEA (56.6%) [16]. This stems from the high cooling rates inherent to LDED, which kinetically restrict atomic diffusion during solidification, enabling the B2 phase to retain more Cr content. The sluggish diffusion effect in HEAs contributes to a low interdiffusion coefficient, thereby promoting a higher Cr concentration within the B2 phase. The elevated Cr content is expected to increase the work function of the LDED B2 phase, thereby reducing the contact potential difference between B2 and FCC phases. This reduction directly diminishes the driving force for micro-galvanic corrosion, thereby enhancing the inherent corrosion resistance of the B2 phase and explaining its morphology after localized corrosion.

Subsequent annealing leads to the formation of three additional types of nanoprecipitates, which in turn degrade corrosion resistance. Li et al. demonstrated that L1_2_ phases below a critical size threshold are beneficial to the corrosion resistance of CoCrFeNiAlTi HEAs; upon complete dissolution of these L1_2_ phases the residual FCC matrix rapidly repassivates, yielding a uniform, Cr-enriched passive film [43,44]. Here, given that the L1_2_ nanoprecipitates within the FCC matrix are present in a low-volume fraction, and are further characterized by their small size of less than 10 nm, they play a negligible role in altering the corrosion behavior. In contrast, the B2 phase shows markedly increased corrosion susceptibility after annealing, as clearly revealed by its highly porous remnants or complete dissolution at the bottom of pits (Figure 15c,d). This selective dissolution of the B2 phase can be linked to the annealing-induced precipitation of Cr-rich σ and BCC phases. As illustrated in Figure 9, the *σ* and BCC phases possess significantly higher Cr content than the B2 phase. Their formation generates passivating-element (Cr)-depleted areas in the surrounding B2 phase (down to 3 at.%), which is a well-recognized mechanism for reduced corrosion resistance in a Cr-depleted matrix [30]. Zhang et al. employed SKPFM to show that the (Cr, Fe)-enriched BCC phase exhibits a higher work function compared to both B2 and FCC, indicating that the Volta potential of the B2 phase is lower, and a correspondingly stronger anodic tendency is manifested relative to the (Cr, Fe)-enriched BCC phase [26]. The potential drop induced by Cr depletion probably facilitates micro-galvanic coupling between the B2 phase and the adjacent *σ*, BCC, and FCC phases, with the B2 phase being the anodic constituent. Consequently, upon passive film breakdown, the B2 phase undergoes preferential dissolution, leaving behind the more stable σ and BCC phases.

Upon the formation of a stable pit, the metal at the bottom of the pit undergoes active dissolution via an acidification mechanism (considering a common metal *M*):

M → M*^n^*^+^ + *ne*^−^ (1)

M*^n^*^+^ + *n*H_2_O → M(OH)_n_ + *n*H^+^ (2)

Theoretically, the high metallic bond energy of Cr imposes a greater energy barrier for active dissolution. Consequently, FCC, *σ* and BCC phases with elevated Cr content exhibit enhanced resistance to active dissolution in pits relative to the B2 phase in the annealed sample. This effect further accelerates the rapid dissolution of the B2 phase.

Phase composition variations can also influence the chemical constituents of surface passivation films, thereby affecting their protective capacity. According to the classical E-pH diagram (Pourbaix diagram) of principal constituent elements (Al, Cr, Fe, Co, Ni) [44,45], only Cr_2_O_3_, Al_2_O_3_, and Fe_2_O_3_ are thermodynamically stable in the solid state at the open-circuit potential of the present HEA in neutral aqueous solution. Our XPS results provide partial support for this finding, revealing the formation of a passive film enriched in Cr and Fe. Previous studies have demonstrated that Cr_2_O_3_ and/or Al_2_O_3_ form the primary constituents of the protective passive film, effectively protecting HEAs against corrosion [30,46,47]. However, Al_2_O_3_ offers inferior resistance relative to Cr_2_O_3_ due to its inherently porous structure [30]. Here, the Al-rich but Cr-depleted B2 phase develops passivation films with reduced Cr_2_O_3_ but elevated Al_2_O_3_ compared to the FCC phase for the LDED sample. Zhou et al. confirmed the high Al_2_O_3_ content of passivation film on the surface of the B2 phase in the AlCoCrFeNi_2.1_ HEA through TEM-EDS analysis [25]. This compositional disparity renders passivation films in the surface of the FCC phase more protective than the B2 phase. Therefore, Cl^−^ preferentially adsorbs onto the passive film of the B2 phase, leading to its weakening and eventual disruption, thereby initiating corrosion. This corrosion typically originates at the FCC/B2 interface, where the Cr-depleted region between B2 and FCC phases serves as a diffusion pathway for Cl^−^ ions. Following annealing, the precipitated *σ* and BCC phases (both Cr-rich but Al-depleted) within the B2 phase further deplete Cr while enriching Al. This elemental redistribution significantly reduces the Cr_2_O_3_ content in the passivation films of the B2 phase, thereby weakening its protective capacity. Consequently, an inhomogeneous passive film is developed on the surface of the annealed sample, together with the reduced overall Cr_2_O_3_ content detected by XPS (Figure 13), thereby compromising its overall corrosion resistance.

**Figure 17:**
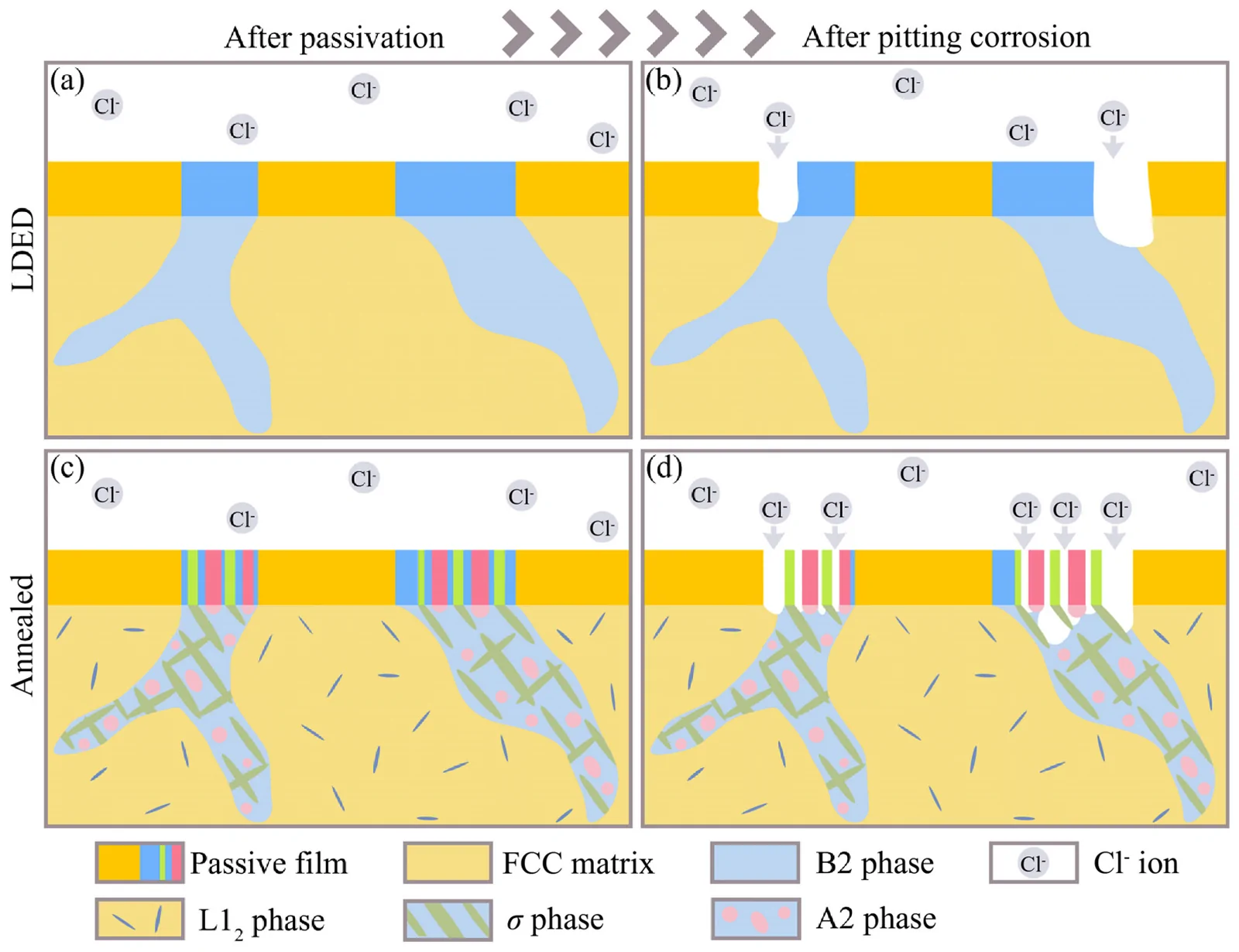
Schematic of the corrosion mechanism of (**a**,**b**) the LDED and (**c**,**d**) annealed samples: (**a**,**c**) after passivation (before pitting initiation) and (**b**,**d**) after pitting corrosion. The distinct colors of the passive film indicate its formation on different phases: yellow = FCC phase passive film, blue = B2 phase passive film, red = (Cr, Fe)-enriched BCC phase passive film, and green = *σ* phase passive film.

On the basis of the preceding discussion, schematic representations of the passivation and corrosion mechanisms for the LDED and annealed samples are shown in Figure 17. In the LDED sample, the passive film (deficient in Cr_2_O_3_) developed on the B2 phase is characterized by comparatively reduced protective efficacy relative to the passive film on the FCC phase. Consequently, Cl^−^ preferentially adsorbs onto the B2 phase, leading to its gradual degradation, and subsequent corrosion initiation preferentially at the FCC/B2 interface due to the high Cl^−^ diffusivity along this interface. Upon exposure, the B2 phase behaves anodically in a micro-galvanic couple with the adjacent FCC phase, inducing preferential dissolution of the B2 phase and subsequent corrosion of the FCC phase. This process ultimately leads to the development of a stable pit. For the annealed sample, the precipitation of σ and BCC phases deteriorates the protective capacity of the passive film on the B2 phase, rendering it highly susceptible to Cl^−^ attack. This precipitation also lowers the potential of the B2 phase through depletion of Cr, thereby facilitating the formation of strong micro-galvanic cells between the B2 phase and the surrounding *σ*, BCC, and FCC phases. With the B2 phase serving as the anode, rapid dissolution occurs, promoting the initiation and stabilization of pits. Consequently, the degraded corrosion resistance of the B2 phase is a primary factor accounting for the diminished overall pitting corrosion resistance observed in the annealed sample.

## 5. Conclusions

(1) The Al_0.5_CoCrFeNi HEA fabricated by LDED exhibits a dendritic microstructure comprising an FCC matrix and an (Al, Ni)-enriched B2 phase. Notably, the Cr content in the B2 phase, albeit higher than that in conventionally manufactured counterparts, remains inferior to the FCC matrix. Subsequent annealing facilitates the precipitation of (Al, Ni)-rich L1_2_ nanophases within the FCC matrix, while triggering the formation of (Cr, Fe, Co)-enriched *σ* and (Cr, Fe)-rich BCC nanoprecipitates within the B2 phase.

(2) Electrochemical measurements demonstrate that both LDED and annealed specimens exhibit intrinsic self-passivation capability. However, the annealed samples display significantly degraded pitting corrosion resistance and repassivation capability relative to their LDED counterparts, as evidenced by reduced pitting potential and repassivation potential.

(3) The precipitation of σ and BCC phases induces Cr depletion concomitant with Al enrichment in the B2 phase. This elemental repartitioning compromises the protective capability of the passive film on the surface of the B2 phase, exacerbates micro-galvanic corrosion susceptibility, and consequently diminishes the corrosion resistance of the annealed HEA.

## Figures and Tables

**Table 1:** The EDS results of the selected points.

Sample	Phase	Point	at.%				
			Al	Co	Cr	Fe	Ni
LDED	B2	A	15.83	19.96	21.53	18.54	24.12
LDED	FCC	B	11.01	21.02	23.90	22.82	21.23
Annealed	FCC	C	12.75	20.52	22.34	21.86	22.51
Annealed	L1_2_	D	17.28	21.10	19.01	18.67	23.92
Annealed	*σ*	E	1.46	31.48	24.24	25.50	17.29
Annealed	*σ*	F	2.31	30.79	27.19	25.43	14.26

**Table 2:** Electrochemical parameters derived from the potentiodynamic polarization curves in this work and recently developed AlCoCrFeNi alloys.

	*I_corr_* (A/cm^2^)	*E_ocp_* (mV)	*E_corr_* (mV)	*E_pit_* (mV)
27SiMn steel	3.98 × 10^−6^ (±1.76)	−623 ± 10	−388 ± 12	—
LDED	1.40 × 10^−7^ (±0.12)	−211 ± 15	−224 ± 21	254 ± 18
Annealed	1.74 × 10^−7^ (±0.34)	−228 ± 10	−247 ± 12	165 ± 23
AlCoCrFeNi [31]	1.45 × 10^−6^		−355	112
Al_0.3_CoCrFeNi [16]	8.35 × 10^−8^ (±0.03)		−195 ± 8	460 ± 18
Al_0.5_CoCrFeNi [16]	2.52 × 10^−7^ (±0.05)		−225 ± 14	385 ± 36
Al_0.7_CoCrFeNi [16]	4.29 × 10^−7^ (±0.05)		−275 ± 9	52 ± 12
AlCoCrFeNi [32]	8.92 × 10^−6^	−292	−285	245
AlCoCrFeNi [33]	2.21 × 10^−5^	−744	−941	
AlCoCrFeNiV [34]	9.30 × 10^−7^ (±0.06)		−52 ± 22	449 ± 24
AlCo_0.5_CrFeNiV_0.5_ [34]	1.59 × 10^−6^ (±0.86)		−124 ± 45	452 ± 74
AlCoCrFeNi [35]	8.50 × 10^−7^		−484	74
AlCoCrFeNiMn_0.5_ [35]	5.99 × 10^−7^		−354	88
AlCoCrFeNi [36]	2.29 × 10^−5^		−616	
AlCoCrFeNiTiSi [36]	2.35 × 10^−6^	−439	−415	

## Data Availability

The original contributions presented in this study are included in the article. Further inquiries can be directed to the corresponding authors.

## References

[1] Sohail Y., Zhang C., Xue D., Zhang J., Zhang D., Gao S., Yang Y., Fan X., Zhang H., Liu G. (2025). Machine-learning design of ductile FeNiCoAlTa alloys with high strength. Nature.

[2] Huo W., Wang S., Dominguez-Gutierrez F.J., Ren K., Kurpaska Ł., Fang F., Papanikolaou S., Kim H.S., Jiang J. (2023). High-entropy materials for electrocatalytic applications: A review of first principles modeling and simulations. Mater. Res. Lett..

[3] Tang Y., Wang R., Xiao B., Zhang Z., Li S., Qiao J., Bai S., Zhang Y., Liaw P.K. (2023). A review on the dynamic-mechanical behaviors of high-entropy alloys. Prog. Mater. Sci..

[4] Haftlang F., Kim H.S. (2021). A perspective on precipitation-hardening high-entropy alloys fabricated by additive manufacturing. Mater. Des..

[5] Zhao Y., Fu X., Lu Y., Yu Q. (2022). Achieving high strength and deformability in a high entropy alloy containing the multiscale phase structure. Mater. Today Nano.

[6] Khan M.A., Brechtl J., Jingyuan L., Radhika N., Zhang Y., Liaw P.K., Liao W.-B., Afifi M.A. (2025). High strength and ductility in a lightweight AlTiNbZrTa refractory high-entropy alloy enabled by nanophase precipitation and solute segregation. Matter.

[7] Yin Y., Wang H., Tan Q., Sun Q., Wu Y., Xu S., Zhao Y., Li M., Liao X., Huang H. (2025). A new route to bulk nanostructured multiphase alloys with ultrahigh hardness. J. Mater. Sci. Technol..

[8] Fu Z., Jiang L., Wardini J.L., MacDonald B.E., Wen H., Xiong W., Zhang D., Zhou Y., Rupert T.J., Chen W. (2018). A high-entropy alloy with hierarchical nanoprecipitates and ultrahigh strength. Sci. Adv..

[9] Shi P., Ren W., Zheng T., Ren Z., Hou X., Peng J., Hu P., Gao Y., Zhong Y., Liaw P.K. (2019). Enhanced strength–ductility synergy in ultrafine-grained eutectic high-entropy alloys by inheriting microstructural lamellae. Nat. Commun..

[10] Gwalani B., Soni V., Lee M., Mantri S., Ren Y., Banerjee R. (2017). Optimizing the coupled effects of Hall-Petch and precipitation strengthening in a Al_0.3_CoCrFeNi high entropy alloy. Mater. Des..

[11] Lou Y., Wang Y., Zhang K., Li J., Wu Y., Gao D., Li Y., Wang H., Shen Z., Zeng X. (2026). Dissolution-driven phase transformation and precipitation in a FeCrNiAl dual-phase HEA exposed to oxygen-deficient LBE. Corros. Sci..

[12] Zhang C., Zhang F., Diao H., Gao M.C., Tang Z., Poplawsky J.D., Liaw P.K. (2016). Understanding phase stability of Al-Co-Cr-Fe-Ni high entropy alloys. Mater. Des..

[13] Bloomfield M., Christofidou K., Mignanelli P., Reponen A.-P., Stone H., Jones N. (2022). Phase stability of the Al CrFeCoNi alloy system. J. Alloys Compd..

[14] Wang W.-R., Wang W.-L., Yeh J.-Y. (2014). Phases, microstructure and mechanical properties of AlxCoCrFeNi high-entropy alloys at elevated temperatures. J. Alloys Compd..

[15] Shi Y., Collins L., Feng R., Zhang C., Balke N., Liaw P.K., Yang B. (2018). Homogenization of Al CoCrFeNi high-entropy alloys with improved corrosion resistance. Corros. Sci..

[16] Shi Y., Yang B., Xie X., Brechtl J., Dahmen K.A., Liaw P.K. (2017). Corrosion of Al CoCrFeNi high-entropy alloys: Al-content and potential scan-rate dependent pitting behavior. Corros. Sci..

[17] Huang X., Zhao Q., Zhan Z., Liu J., Liao X., Deng J., Wei L., Li X. (2023). Effect of Al addition on the corrosion behavior of Al CoCrFeNi high entropy alloys in supercritical water. Corros. Sci..

[18] Liu Y., Sun J., Fu Y., Xu B., Li B., Xu S., Huang P., Cheng J., Han Y., Han J. (2021). Tuning strength-ductility combination on selective laser melted 316L stainless steel through gradient heterogeneous structure. Addit. Manuf..

[19] ZLiu Z., Zhao D., Wang P., Yan M., Yang C., Chen Z., Lu J., Lu Z. (2022). Additive manufacturing of metals: Microstructure evolution and multistage control. J. Mater. Sci. Technol..

[20] Moghaddam A.O., Shaburova N.A., Samodurova M.N., Abdollahzadeh A., Trofimov E.A. (2021). Additive manufacturing of high entropy alloys: A practical review. J. Mater. Sci. Technol..

[21] Tian Y., Lu Y., Narayan R.L. (2024). Optimization of tensile properties and anisotropy in a cryogenically treated laser additively manufactured high entropy alloy. Int. J. Plast..

[22] Xu L., Bai Y., Guo Y., Ren C., Tan X., Jia Y., Wang G., Wang H. (2024). Abnormal mechanochemical effect in ultraprecision machining of an additively manufactured precipitation-strengthened high-entropy alloy. J. Mater. Sci. Technol..

[23] Sathiyamoorthi P., Kim H.S. (2022). High-entropy alloys with heterogeneous microstructure: Processing and mechanical properties. Prog. Mater. Sci..

[24] Guo B., Yang Z., Wu Q., Xu C., Cui D., Jia Y., Wang L., Li J., Wang Z., Lin X. (2024). Multi-scale annealing twins generate superior ductility in an additively manufactured high-strength medium entropy alloy. Int. J. Plast..

[25] Zhou L., Zhang J., Feng G., Chen Y., Xie Y., Zhang J., Li G., Zhou T., Zhou Y., Duan F. (2025). Homogenization of AlCoCrFeNi2.1 eutectic high entropy with improved corrosion resistance fabricated by selective laser melting. Corros. Sci..

[26] Zhang C., Huang L., Li S., Li K., Lu S., Li J. (2023). Improved corrosion resistance of laser melting deposited CoCrFeNi-series high-entropy alloys by Al addition. Corros. Sci..

[27] Wei L., Qin W. (2022). Corrosion mechanism of eutectic high-entropy alloy induced by micro-galvanic corrosion in sulfuric acid solution. Corros. Sci..

[28] Tsai M.-H., Yeh J.-H. (2014). High-Entropy Alloys: A Critical Review. Mater. Res. Lett..

[29] Rao J.C., Diao H.Y., Ocelík V., Vainchtein D., Zhang C., Kuo C., Tang Z., Guo W., Poplawsky J., Zhou Y. (2017). Secondary phases in AlxCoCrFeNi high-entropy alloys: An in-situ TEM heating study and thermodynamic appraisal. Acta Mater..

[30] Duan X., Han T., Guan X., Wang Y., Su H., Ming K., Wang J., Zheng S. (2023). Cooperative effect of Cr and Al elements on passivation enhancement of eutectic high-entropy alloy AlCoCrFeNi_2.1_ with precipitates. J. Mater. Sci. Technol..

[31] Kuwabara K., Shiratori H., Fujieda T., Yamanaka K., Koizumi Y., Chiba A. (2018). Mechanical and corrosion properties of AlCoCrFeNi high-entropy alloy fabricated with selective electron beam melting. Addit. Manuf..

[32] Nanthini M., Jagajeevan V. (2026). Multifunctional (AlCoCrFeNi)_65_Mo_15_N_5_W_5_ high-entropy alloy coating with simultaneous enhancement in corrosion, Wear and anti-biofouling performance for marine applications. Surf. Coat. Tech..

[33] Zhang H., Qiao M., Yue X.-J., Zhang P., Sun W., Ni X., Wang W., Yan X., Zhang D. (2026). Effect of rare-earth particle doping on the corrosion performance of the AlCoCrFeNi coatings prepared by extremely high-speed laser cladding. J. Alloy. Compd..

[34] Souto C.A., Mirhan A.L.R., Yao J.-Y., Serrano L.B., Travessa D.N., Cardoso K.R. (2024). Microstructural characteristics and corrosion behaviour of AlCoCrFeNi high-entropy alloys containing Vanadium. Electrochim. Acta.

[35] Zhang D., Li Q., Chang C., Zhang G., Ma X., Sun R. (2025). Effects of Mn addition on wear and corrosion resistances of AlCoCrFeNi high-entropy alloy coating sprayed by HVOF. J. Mater. Res. Technol..

[36] Li Z., Mei K., Dong J., Yang Y., Sun J., Luo Z. (2024). An investigation on the wear and corrosion resistance of AlCoCrFeNi high-entropy alloy coatings enhanced by Ti and Si. Surf. Coat. Tech..

[37] Li T., Wu J., Frankel G.S. (2021). Localized corrosion: Passive film breakdown vs. Pit growth stability, Part VI: Pit dissolution kinetics of different alloys and a model for pitting and repassivation potentials. Corros. Sci..

[38] Sun J., Sun Q., Liu Y., Li B., Zhang Z., Xu B., Xu S., Han Y., Qiao Y., Han J. (2022). Improving corrosion resistance of selective laser melted 316L stainless steel through ultrasonic severe surface rolling. J. Mater. Res. Technol..

[39] Kang K., Li A., Zhang B., Zhang J., Xu M., Huang D., Liu S., Jiang Y., Li G. (2026). Fabrication of non-equiatomic CoCrFeNi_2_ HEAs with exceptional strength and corrosion resistance. J. Mater. Sci. Technol..

[40] Qiu Y., Thomas S., Gibson M.A., Fraser H.L., Birbilis N. (2017). Corrosion of high entropy alloys. npj Mater. Degrad..

[41] Kong D., Dong C., Wei S., Ni X., Zhang L., Li R., Wang L., Man C., Li X. (2021). About metastable cellular structure in additively manufactured austenitic stainless steels. Addit. Manuf..

[42] Gwalani B., Gorsse S., Choudhuri D., Styles M., Zheng Y., Mishra R.S., Banerjee R. (2018). Modifying transformation pathways in high entropy alloys or complex concentrated alloys via thermo-mechanical processing. Acta Mater..

[43] Li T., Wang D., Cui J., Li N., Zhang S., Wang J. (2025). Size-dependent pitting corrosion behavior for L12-strengthened high-entropy alloys. Corros. Sci..

[44] Li T., Wang D., Cui J., Wang Q., Zhang S., Wang J. (2025). Enhanced pitting resistance for high-entropy alloys by precipitating nano-sized L12-strengthened phase. J. Mater. Sci. Technol..

[45] Chen M., Jia Z., Wang Z., Xiang C., Pu J., Sun G., Han E.-H. (2024). Insights into the passivity and electrochemistry of CoCrFeMnNi high entropy alloy fabricated by underwater laser direct metal deposition. Corros. Sci..

[46] Zengin H., Greul A., Turan M.E., Duchoslav J., Mardare A.I., Hassel A.W. (2025). Corrosion and passivation behaviour of as-cast and heat-treated AlCoCrFeNiX_0.5_ (X=Mo, Ta) high entropy alloys in 3.5 wt.% NaCl solution. Electrochim. Acta.

[47] Zhang J.Y., Zhou Y.H., Ren Z.Q., Luan J.H., Zhao Y.L., Yang T. (2024). The influence of microstructural evolutions on electrochemical corrosion and passive behavior in precipitation-strengthened high-entropy alloys. Corros. Sci..

